# Dapagliflozin Protects Methamphetamine-Induced Cardiomyopathy by Alleviating Mitochondrial Damage and Reducing Cardiac Function Decline in a Mouse Model

**DOI:** 10.3389/fphar.2022.925276

**Published:** 2022-07-07

**Authors:** Shanqing He, Yajun Yao, Nan Yang, Youcheng Wang, Dishiwen Liu, Zhen Cao, Huiyu Chen, Yuntao Fu, Mei Yang, Songjun Wang, Guangjie He, Qingyan Zhao

**Affiliations:** ^1^ Department of Cardiology, Renmin Hospital of Wuhan University, Wuhan, China; ^2^ Cardiovascular Research Institute of Wuhan University, Wuhan, China; ^3^ Hubei Key Laboratory of Cardiology, Wuhan, China; ^4^ School of Forensic Medicine, Xinxiang Medical University, Xinxiang, China; ^5^ Hebei Key Laboratory of Forensic Medicine, Hebei Medical University, Shijiazhuang, China

**Keywords:** cardiomyopathy, methamphetamine, dapagliflozin, mitochondrial, apoptosis

## Abstract

**Background:** Methamphetamine (METH)-induced cardiovascular toxicity has been attributed to its destructive effect on mitochondrial function at least to some extent. Previous studies highlighted the benefits of dapagliflozin (DAPA) on the cardiovascular system, but the response of METH-induced cardiomyopathy to DAPA is never addressed before. The present study aimed to investigate the potential ability of DAPA in preventing METH-induced cardiomyopathy.

**Materials and Methods:** C57BL/6 mice were randomly divided into control group (*n* = 24), METH group (*n* = 24), and METH + DAPA group (*n* = 24). The METH-induced cardiomyopathy group received intraperitoneal METH injections at gradually increasing doses thrice weekly for 14 weeks. Mice in the METH + DAPA group were simultaneously treated with DAPA 1 mg/kg/day by intragastric administration. Echocardiography was performed to assess cardiac function. Reactive oxygen species (ROS), JC-1, and terminal deoxynucleotidyl transferase dUTP nick-end labeling (TUNEL) assays were performed to evaluate oxidative stress, mitochondrial damage, and apoptosis, respectively. Mitochondrial and apoptosis-related protein expression was measured by western blotting.

**Results:** Mice exposed to METH exhibited reduced cardiac function (left ventricular ejection fraction [LVEF]: 56.51 ± 6.49 vs. 73.62 ± 1.42, *p* < 0.01), fibrotic remodeling, and mitochondrial dysfunction, leading to apoptosis (apoptotic cells%: 7.4 ± 1.3 vs. 1.3 ± 0.5, *p* < 0.01). DAPA significantly reduced mitochondrial dynamics and function, ROS, apoptosis (apoptotic cells%: 2.4 ± 0.8 vs. 7.4 ± 1.3, *p* < 0.01), cardiac function decline (LVEF: 70.99 ± 4.936 vs. 56.51 ± 6.49, *p* < 0.01), and fibrotic remodeling. These results indicated that DAPA could be considered as an effective therapeutic agent in the protection against METH-associated cardiomyopathy.

**Conclusion:** DAPA protects against METH-induced cardiomyopathy in mice by decreasing mitochondrial damage and apoptosis.

## Background

Methamphetamine (METH) is a highly addictive sympathomimetic stimulant, and its illicit use is a global concern. Several clinical and autopsy reports associate METH with cardiomyopathy, arrhythmias, myocarditis, and sudden death (Karch et al., 1999; Wijetunga et al., 2003; Reddy et al., 2021). METH increases oxidative stress and apoptosis, alters mitochondrial function, and affects cardiac contractility (Lord et al., 2010; Jafari Giv, 2017; Abdullah et al., 2020). It significantly increased the production of reactive oxygen species (ROS) in a METH-exposed rat model, leading to higher nitrification of cardiac contractile proteins and mitochondrial proteins (Lord et al., 2010). In another mouse study, METH decreased mitochondrial respiration and the expression of the mitochondrial fission regulatory protein FIS1 in the heart (Abdullah et al., 2020). Mitochondrion-related endothelial cell apoptosis is also linked to the cardiotoxic effects of METH (Cai et al., 2016). In particular, METH targets the Bcl-associated X (Bax)/B-cell lymphoma (Bcl-X)-caspase3 axis to induce mitochondrial apoptosis (Liou et al., 2014).

Dapagliflozin (DAPA) is a sodium-glucose cotransporter 2 inhibitor (SGLT2I) developed as an oral hypoglycemic agent (Garcia-Ropero et al., 2018). A clinical trial showed that SGLT2I reduced cardiovascular morbidity in patients with type 2 diabetes mellitus who are at a high risk of developing cardiovascular events (Fitchett et al., 2019). SGLT2 inhibitors in nondiabetic mice improve vascular endothelial function, ameliorate myocardial fibrosis, decrease oxidative stress, and enhance cardiac systolic function (Lin et al., 2014; Joubert et al., 2017; Lee et al., 2018). In addition, DAPA attenuates cardiac fibrosis in infarcted rat hearts by regulating macrophage phenotype switching through the ROS pathway (Lee et al., 2017). Furthermore, DAPA reduced cardiac damage by suppressing oxidative stress and protecting cardiac mitochondrial function in a cardiac reperfusion injury rat model (Tanajak et al., 2018). Despite its potential benefits in cardiac function, the cardioprotective effects of DAPA against METH-induced damage have never been investigated. Therefore, in this study, METH-induced cardiomyopathy was established through thrice-weekly intraperitoneal METH injections for 14 weeks to observe whether DAPA can alleviate the decline in cardiac function induced by METH. Further, the potential underlying molecular mechanisms have been evaluated.

## Materials and Methods

### Animal Treatments

**FIGURE 1:**
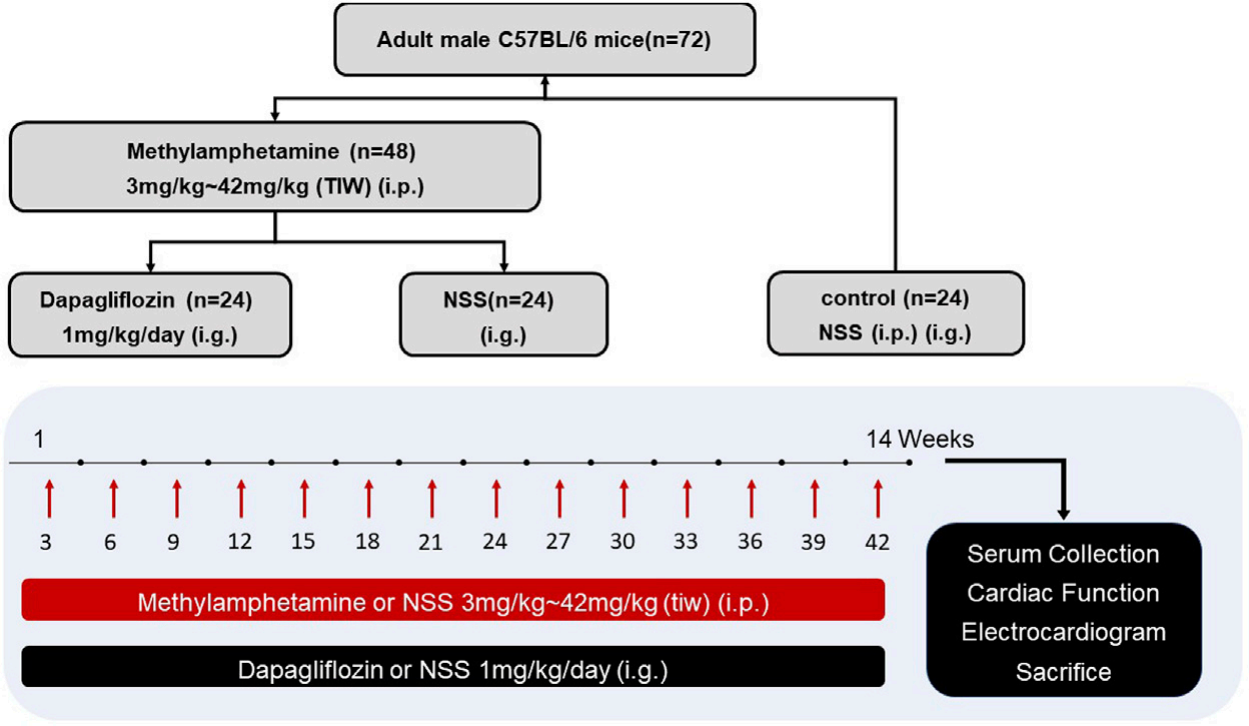
Experimental protocol. Diagram illustrating the experimental protocol of this study. i.p., intraperitoneal injection; NSS, normal saline solution; i.g., intragastric injection; TIW, three times a week.

C57BL/6 mice (18–20 g; 8–10 weeks old) were obtained from Beijing Vital River Laboratory Animal Technology Co., Ltd. METH was purchased from the National Institutes for Food and Drug Control. Only male mice were used in this study since males are sensitive to METH toxicity (Marcinko et al., 2019). All mice were fed with standard chow and water and were housed under a 12-hour light/dark cycle. The mice were randomly divided into control (*n* = 24), METH (*n* = 24), and METH + DAPA groups (*n* = 24) as shown in Figure 1. The METH group and the MATH + DAPA group received thrice-weekly intraperitoneal METH injections in the left upper quadrant of the abdomen using a 1 ml syringe. In contrast, the control group received injections of normal saline solution (aqueous 0.9% sodium chloride solution). METH was dissolved in normal saline solution, and the dose was gradually increased at a rate of 3 mg per week for the entire 14-week study, reaching the final dose of 42 mg/kg. The METH + DAPA group also received an intragastric administration of DAPA 1 mg/kg/day. All experimental procedures were approved by the Ethics Committee of Xinxiang Medical University. This study complies with the institutional guidelines for the care and use of experimental animals.

### Echocardiography

All echocardiography experiments were performed on anesthetized mice (*n* = 8 in each group). Briefly, mice were anesthetized under isoflurane (2–3% for the induction of anesthesia and 1% for its maintenance), and the echocardiographic assessment was conducted using the Visual Sonics Vevo 2100 echocardiographic system, equipped with a 30 MHz transducer (FUJIFILM Visual Sonics Inc., Toronto, Canada). A 2-dimensional (2-D) parasternal short-axis view of the left ventricle (LV) was obtained at the level of the papillary muscle. The cardiac function and dimensions were measured, including the left ventricular internal diameter at end systole (LVIDs), left ventricular internal diameter at end diastole (LVIDd), left ventricular posterior wall thickness at end systole (LVPWs), and left ventricular posterior wall thickness at end diastole (LVPWd). Left ventricular percent fractional shortening [%LVFS = (LVID; d-LVID; s)/LVID; d×100%] and left ventricular percent ejection fraction [%LVEF = (LV Vol; d-LV Vol; s)/LV Vol; d×100%] were calculated as systolic cardiac functions.

The body temperature was maintained at 36–37.0°C to avoid the confounding effects of hypothermia. All echocardiographic measurements were performed in a blinded manner and in compliance with the American Society of Echocardiography guidelines (Lang et al., 2015). Three independent measurements were obtained from each animal, and the results were reported as the average of three cardiac cycles.

### Histopathological Analysis

Immediately after each euthanasia, the heart was removed, and the ventricle was dissected. The LV wall samples were fixed in 4% paraformaldehyde and embedded in paraffin. Sections cut from heart paraffin blocks were stained with Masson’s trichrome to determine the degree of cardiac fibrosis within the collagen volume fraction (CVF) since collagen is stained blue using this staining. Three heart samples from different animals in each group were randomly selected and used to quantify the fibrotic area in three different fields at ×400 magnification. The results were analyzed using Image-Pro Plus 6.0 software.

### Microscopy for the Evaluation of Reactive Oxygen Species Generation

Immediately after each euthanasia, the heart was removed, and the ventricle was dissected. The LV wall samples were cooled with Drikold and stored at −80°C. The chilled ventricle tissues were sectioned using a cryostat microtome, and then the ROS detection kit (DHE probe, KGAF019, KeyGEN) was used to detect the amount of ROS. Nuclei were subsequently stained with DAPI. The medium was removed after 30 min of incubation at 37°C, and the specimens were examined under a LEICA DMIL (IX51, OLYMPUS) microscope. Three samples from different animals in each group were randomly selected and used to quantify ROS generation in three different fields at ×400 magnification. ROS staining was quantified as DHE-positive cells/total cells.

### Measurement of Mitochondrial Membrane Potential

The refrigerated ventricle tissues were thawed to room temperature, and the JC-1 ΔΨm detection kit (C2006, Beyotime Biotechnology) was used to evaluate the mitochondrial membrane potential. The specimens were then examined under a LEICA DMIL microscope. Three samples in each group from different animals were randomly selected and used to quantify mitochondrial membrane potential in three different fields at ×400 magnification. The results were quantified as red optical density/green optical density and analyzed using Image-Pro Plus 6.0 software.

### Transmission Electron Microscopy

Mice LV heart sections were fixed in 2% glutaraldehyde and post-fixed in 1% OsO_4_ for sectioning. Multiple sections were counterstained with uranium and lead salts, followed by an examination with a Hitachi transmission electron microscope. Images were acquired using a Zoom-1 HC-1 digital camera.

### Enzyme-Linked Immunosorbent Assay

Blood samples from the tail vein were obtained at the end of 14 weeks and centrifuged at 3000 rpm for 10 min at 4°C to extract the plasma. These samples were temporarily stored at −80°C until the assay. Afterward, plasma samples from the control, METH, and METH + DAPA groups were subjected to ELISA for noradrenaline (NE) levels (ELK Biotechnology, China) and the serum levels of B-type natriuretic peptide (BNP) (Abcam Ab193694). All procedures were performed according to the manufacturer’s instructions.

### Terminal Deoxynucleotidyl Transferase dUTP Nick-End Labeling Assay

The paraffin-embedded sections were deparaffinized with xylene, then rehydrated using different ethanol gradients. The sections were treated with citric acid antigen repair buffer and blocked with bovine serum albumin (Servicebio. G5001). Next, they were incubated with the primary anti-α-actinin antibody (Servicebio, GB11555, 1:200), incubated overnight, and then incubated with the secondary antibody (Servicebio, GB21303, 1:300). TUNEL assay was used to detect apoptotic cells using an *In Situ* Apoptosis Detection Kit (Roche, 11684817910). The kit was used together with DAPI nuclear labeling. More than three sections from different samples were randomly selected to quantify the percentage of apoptotic cells.

### Western Blotting

Western blotting was performed to determine the expression of the following proteins in the ventricle tissues (*n* = 8 in each group): fission1 (FIS1) (Bioss, bs7646R), mitofusin2 (MFN2) (ab124773, Abcam), Bcl-Associated X (Bax) (#2772, CST), B-cell lymphoma-2 (Bcl-2) (ab196495, Abcam), cleaved caspase3 (AF7022, Affbiotech), caspase9 (ab198061, Abcam), cytochrome C (Cyt-c) (133504, Abcam), and inhibitor of apoptosis 1 (c-IAP1) (Bioss, bs4262R). The membrane was blocked using 5% nonfat dry milk in Tris-buffered saline with Tween 20 (TBST) for 1 h and incubated with the primary antibody overnight at 4°C. Then, the membrane was washed thrice in TBST, incubated with the secondary antibody for 1 h at 37°C, and imaged using Immun-Star horseradish peroxidase substrate. The relative expression of the proteins was determined using the image analyzer software (AlphaEase FC, San Leandro, CA, United States).

### Immunocytochemistry

Ventricle tissue sections were incubated with primary antibodies against cleaved caspase3 (Servicebio, GB11532) and GRP78 (Servicebio, GB11098), and the nuclei were stained with DAPI. The results were analyzed by Image-Pro Plus 6.0 software. More than three ventricle sections from different animals were randomly selected to quantify the expression of cleaved caspase3 and GRP78. The mean optical density of the protein was calculated as follows: sum of the optical density values/total area.

### Statistical Analysis

Statistical analysis was performed using GraphPad Prism 8 software. One-way ANOVA was used to compare the mean values of continuous variables in the three groups. Values are presented as the mean ± standard deviation. All the statistical tests were two-sided, and a value of *p* < 0.05 was considered statistically significant.

## Results

### Dapagliflozin Improves Cardiac Function and Structure in Mice Exposed to Methamphetamine

**FIGURE 2:**
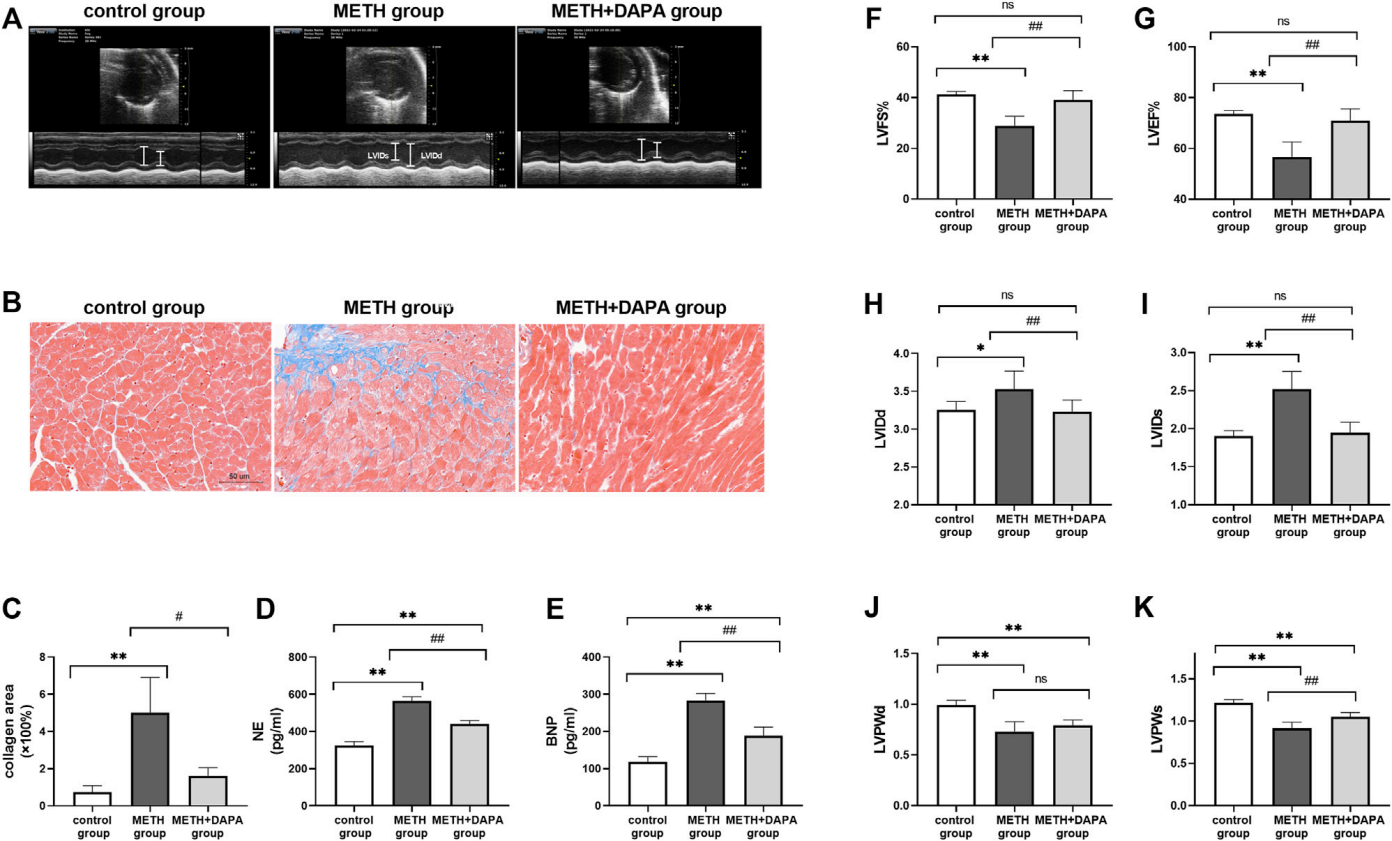
Echocardiography and cardiac remodeling. **(A)** Representative echocardiography in the three groups; LVIDd and LVIDs are shown in white lines. **(B)** Representative Masson’s trichrome in the ventricle of each group. The collagen fibers were stained in blue. **(C)** Quantitative analysis of Masson’s trichrome in each group. **(D)** and **(E)** Serum NE and BNP concentrations in each group. **(F)** and **(G)** %LVEF and %LVFS in each group. **(H)** and **(I)** LVIDs and LVIDd in each group. **(J)** and **(K)** LVPWs and LVPWd in each group. ^*^ *p* < 0.05 vs*.* the control group, ^**^ *p* < 0.01 vs*.* the control group. ^#^ *p* < 0.05 vs*.* the METH group, ^##^ *p* < 0.01 vs*.* the METH group. METH, methamphetamine; DAPA, dapagliflozin; LVEF, left ventricular ejection fraction; LVFS, left ventricular fraction shortening; LVIDd, left ventricular internal diameter at end-diastole; LVIDs, left ventricular internal diameter at end-systolic; LVPWd, left ventricular posterior wall thickness at end diastole; LVPWs, left ventricular posterior wall thickness at end systole; NE, noradrenaline; BNP, B-type natriuretic peptide.

The measurements of LV systolic and diastolic function are shown in Figure 2. Representative echocardiographic images in M-mode at week 14 (Figure 2A) show that mice in the METH group had lower %LVEF (LVEF: 56.51 ± 6.49 vs. 73.62 ± 1.42, *p* < 0.01), %LVFS (LVFS: 28.87 ± 3.78 vs. 41.28 ± 1.17, *p* < 0.01), LVPWs (LVPWs: 0.92 ± 0.07 vs. 1.22 ± 0.04, *p* < 0.01), and LVPWd (LVPWd: 0.73 ± 0.10 vs. 0.99 ± 0.05, *p* < 0.01) and higher levels of LVIDs (LVIDs: 2.52 ± 0.23 vs. 1.91 ± 0.07, *p* < 0.01) and LVIDd (LVIDd: 3.53 ± 0.24 vs. 3.25 ± 0.11, *p* < 0.01) than those in control mice. Mice in the DAPA + METH group had higher %LVFS (LVEF: 70.99 ± 4.936 vs. 56.51 ± 6.49, *p* < 0.01), LVFS (LVFS: 39.12 ± 3.67 vs. 28.87 ± 3.78, *p <* 0.01), and LVPWs (LVPWs: 1.05 ± 0.05 vs. 0.92 ± 0.07, *p* < 0.01) and lower levels of LVIDs (LVIDs: 1.95 ± 0.14 vs. 2.52 ± 0.23, *p* < 0.01) and LVIDd (LVIDd: 3.23 ± 0.15 vs. 3.53 ± 0.24, *p* < 0.01) than those in the METH mice. LVPWd in the METH and DAPA + METH groups did not differ significantly (Figures 2F–K). Although LVPWd and LVPWs of the mice in the DAPA group decreased compared with those in the control group (*p* < 0.01), %LVEF, %LVFS, LVIDd, and LVIDs in the DAPA group were not significantly different compared with those in the control group. In summary, DAPA administration reduced systolic cardiac functional decline caused by METH in these mice.

### Dapagliflozin Reduces Myocardial Injury and Adverse Fibrotic Remodeling Caused by Methamphetamine Exposure

Heart sections stained with Masson’s trichrome stain to evaluate fibrosis and collagen deposition in the myocardium revealed a significant upregulation of the fibrotic area in the METH group compared with the control group (fibrosis%: 5.00 ± 1.90 vs. 0.73 ± 0.35, *p* < 0.01) and downregulation of the percentage of fibrotic area in the METH + DAPA group compared with the METH group (fibrosis%: 1.60 ± 0.46 vs. 5.00 ± 1.90, *p* < 0.05) (Figures 2B,C). In addition, serum NE and BNP levels, markers of heart failure progression, were higher in the METH group than in the control group (NE, ng/mL: 564 ± 23 vs. 324 ± 21, *p* < 0.01; BNP, ng/mL: 283 ± 19 vs. 118 ± 14, *p* < 0.01) and lower in the DAPA + METH group than in the METH group (NE, ng/mL: 439 ± 20 vs. 564 ± 23, *p* < 0.01; BNP, ng/mL: 189 ± 23 vs. 283 ± 19, *p* < 0.01) (Figures 2D,E).

### Dapagliflozin Alleviated Oxidative Stress Induced by Methamphetamine

**FIGURE 3:**
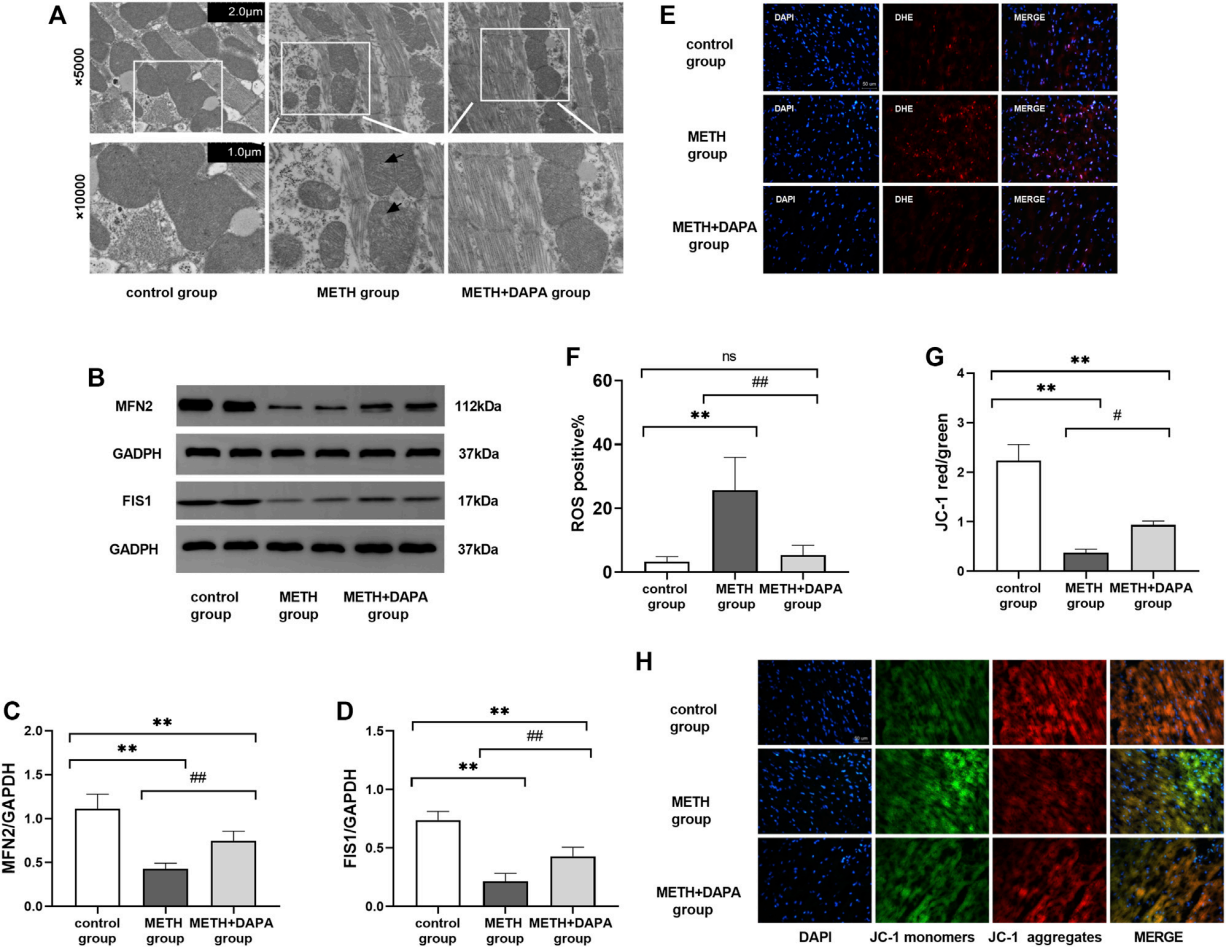
Oxidative stress and mitochondrial damage. **(A)** Representative electron microscope photographs from the three groups. Mice in the METH group showed the rupture of mitochondrial cristae (white arrow) compared with the control group and METH + DAPA group. **(B)** Representative bands of the expression of the mitochondrial protein MFN2 and FIS1 in each group. **(C)** and **(D)** Quantitative analysis of the expression of the mitochondrial protein MFN2 and FIS1 in each group. **(E)** Representative ROS staining in each group. ROS was stained red by DHE. **(F)** Quantitative analysis of ROS staining. ROS staining was quantified in terms of DHE positive cells/total cells. **(G)** Representative figure of JC-1 staining. Red fluorescence intensity represents the number of aggregate mitochondria, while green fluorescence intensity represents the number of monomeric mitochondria. **(H)** Quantitative analysis of JC-1 staining according to the red optical density/green optical density. ^*^ *p* < 0.05 vs*.* the control group, ^**^ *p* < 0.01 vs*.* the control group. *p < 0.05 vs*.* the METH group, ^##^ *p* < 0.01 vs*.* the METH group. ROS, reactive oxygen species; METH, methamphetamine; DAPA, dapagliflozin; ROS, reactive oxygen species; DHE, Dihydroethidium; MFN2, mitochondrial fusion protein 2; FIS1, fission1.

ROS levels in the myocardium were significantly higher in the METH group than in the METH + DAPA group (ROS positive%: 25.1 ± 10.3 vs. 3.3 ± 1.5,*p* < 0.05) and control group (ROS positive%: 25.1 ± 10.3 vs. 5.3 ± 3.0,*p* < 0.05), suggesting that DAPA suppressed ROS production (Figures 3E,F).

### Dapagliflozin Improves Mitochondrial Morphology in Methamphetamine-Exposed Mice

Transmission electron microscopy to study the mitochondrial morphology of myocytes (Figure 3A) showed that METH treatment for 14 weeks ruptured the mitochondrial cristae. However, DAPA significantly decreased METH-induced injury.

Cardiac mitochondrial dynamics include mitochondrial fusion and mitochondrial fission. The level of cardiac MFN2 was significantly lower in the METH group than in the control group (MFN2: 1.11 ± 0.17 vs. 0.43 ± 0.07, *p* < 0.01). DAPA + METH mice had significantly higher MFN2 levels than METH mice (MFN2: 0.43 ± 0.07 vs. 0.74 ± 0.11, *p* < 0.01) (Figure 3C). The representative bands of cardiac mitochondrial MFN2 protein expression are shown in Figure 3B. METH downregulated FIS1 expression (FIS1: 0.21 ± 0.07 vs. 0.74 ± 0.07, *p* < 0.01), but DAPA upregulated its level (FIS1: 0.43 ± 0.08 vs. 0.74 ± 0.07, *p* < 0.01) (Figure 3D). The expression of FIS1 and MFN2 was lower in the DAPA group than in the control group (*p* < 0.01).

JC-1 staining (Figures 3G,H) showed a significantly lower mitochondrial red/green fluorescence intensity ratio in the METH group than in the control group, indicating mitochondrial depolarization. DAPA improved cardiac mitochondrial function as indicated by the increased mitochondrial red/green fluorescence intensity ratio compared with that in the METH group.

### Dapagliflozin Reduces Cardiomyocyte Apoptosis Through the Cytochrome C Mediated Apoptosis Pathway

**FIGURE 4:**
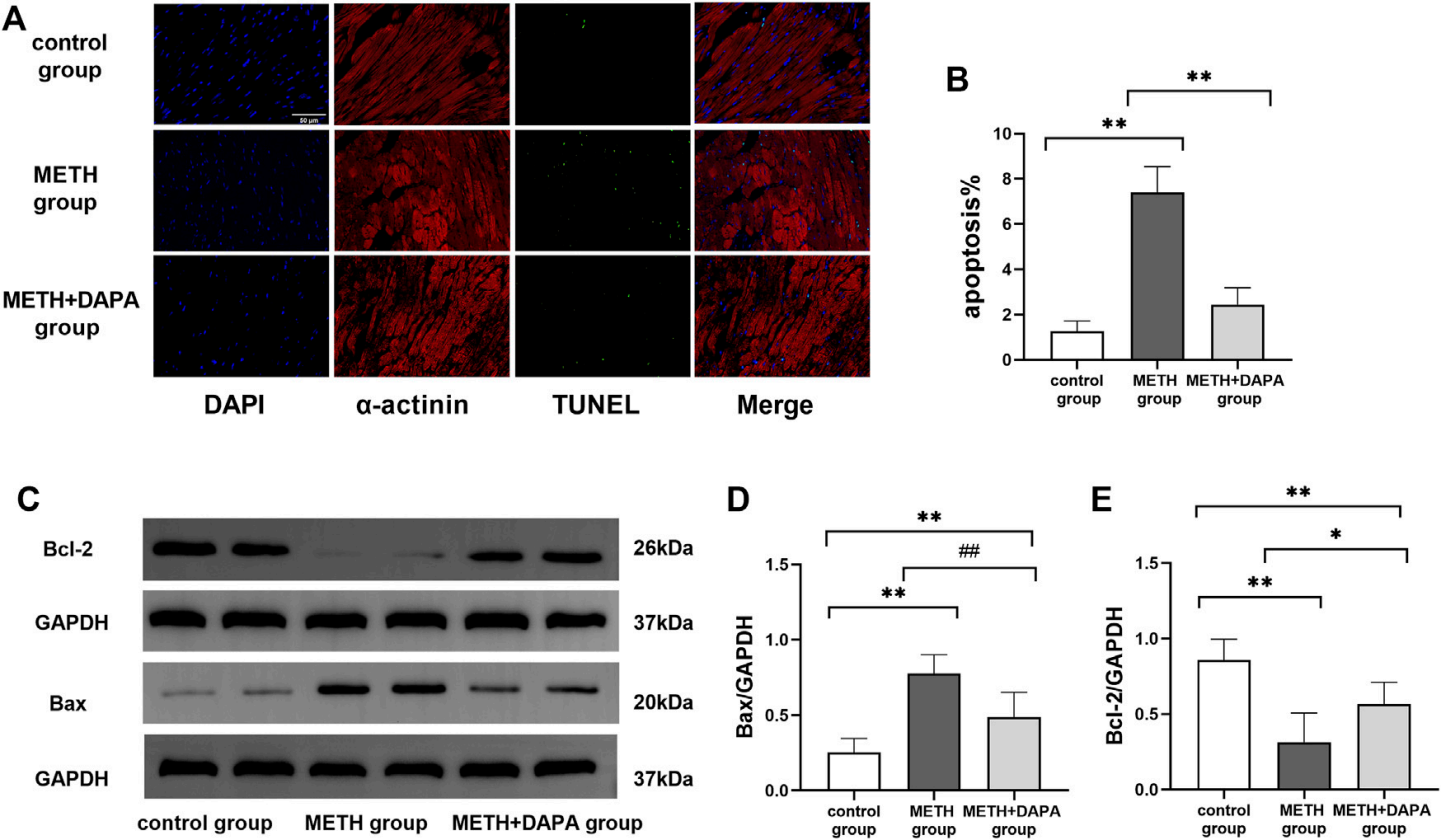
Myocardial apoptosis. **(A)** Representative TUNEL-α-actinin immunofluorescence double staining in each group. The myocardial cells were labeled with α-actinin and stained red, while the apoptotic nuclei were stained green. **(B)** Quantitative analysis of TUNEL staining by FITC/DAPI in each group. **(C)** Representative bands of Bax and Bcl-2 in each group. **(D)** and **(E)** Quantitative analysis of Bax and Bcl-2 expression in each group. ^**^ *p* < 0.01 vs*.* the control group. ^#^ *p* < 0.05 vs*.* the METH group, ^##^ *p* < 0.01 vs*.* the METH group. METH, methamphetamine; DAPA, dapagliflozin; Bax, Bcl-Associated X; Bcl-2, B-cell lymphoma-2.

**FIGURE 5:**
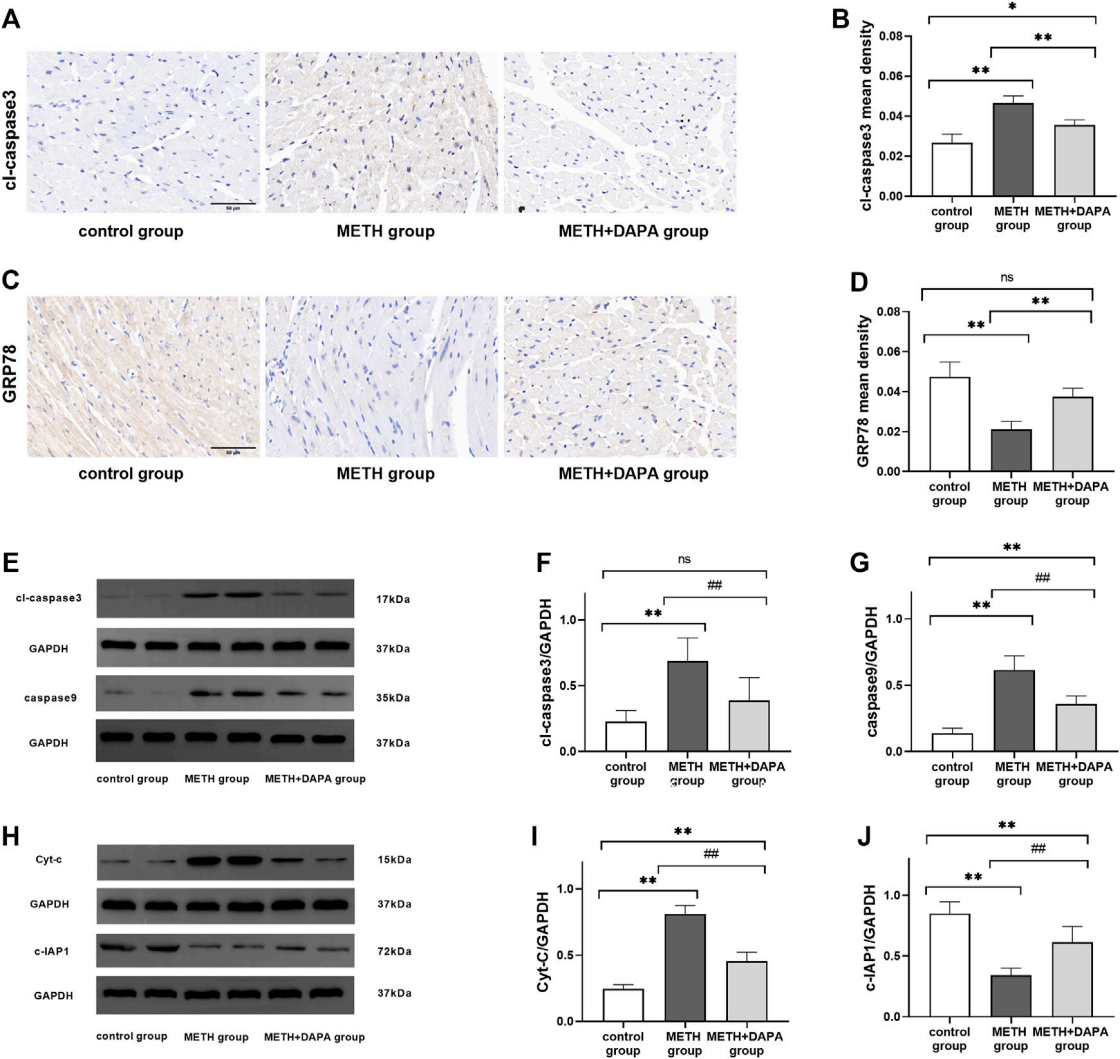
Signaling pathway protein expression. **(A)** Representative image of cleaved caspase3 by immunohistochemistry in each group. **(B)** Quantitative analysis of cleaved caspase3 by immunohistochemistry in each group. **(C)** Representative image of GRP78 by immunohistochemistry in each group. **(D)** Quantitative analysis of GRP78 by immunohistochemistry in each group. **(E)** Representative bands of cleaved caspase3 and caspase9 in each group. **(F)** and **(G)** Quantitative analysis of the expression of cleaved caspase3 and caspase9 in each group. **(H)** Representative bands of Cyt-C, cIAP1 in each group. **(I)** and **(J)** Quantitative analysis of the expression of Cyt-C and cIAP1 in each group. ^*^ *p* < 0.05 vs*.* the control group, ^**^ *p* < 0.01 vs*.* the control group.^#^ *p* < 0.05 vs*.* the METH group, ^##^ *p* < 0.01 vs*.* the METH group. METH, methamphetamine; DAPA, dapagliflozin; cl-caspase3, cleaved caspase3; GRP78, glucose regulated protein; Cyt-C, cytochrome C; cIAP1, inhibitor of apoptosis 1.

Double staining of TUNEL and α-actinin immunofluorescence showed the activation of apoptosis by METH. The percentage of apoptotic myocardial cells in the METH group was significantly higher than that in the control group (apoptotic cells%: 7.4 ± 1.3 vs. 1.3 ± 0.5, *p* < 0.01), but apoptosis was effectively suppressed by DAPA (apoptotic cells%: 2.4 ± 0.8 vs. 7.4 ± 1.3, *p <* 0.01) (Figure 4A). In addition, the expression levels of Bax, Bcl-2, cleaved caspase3, caspase9, c-IAP1 and Cyt-C were evaluated by western blotting. Mice in the METH group had significantly higher expression of Bax (Bax: 0.78 ± 0.13 vs. 0.25 ± 0.09, *p* < 0.01) and lower expression of Bcl-2 (Bcl-2: 0.31 ± 0.20 vs. 0.86 ± 0.14, *p* < 0.01) than mice in the control group. DAPA reduced the expression of cardiac Bax (Bax: 0.49 ± 0.17 vs. 0.78 ± 0.13, *p* < 0.01) while increasing the expression of Bcl-2 (Bcl-2: 0.57 ± 0.14 vs. 0.31 ± 0.20, *p* < 0.05), compared with their expression in the METH group. The expression of Bax increased while the expression of Bcl-2 decreased compared with their expression in the control group (*p* < 0.01) (Figures 4C–E). Mice in the METH group had significantly higher expression of cleaved caspase3 (cleaved caspase3: 0.69 ± 0.18 vs. 0.23 ± 0.09, *p* < 0.01), caspase9 (caspase9: 0.62 ± 0.10 vs. 0.14 ± 0.04, *p* < 0.01), and Cyt-C (Cyt-C: 0.81 ± 0.06 vs. 0.24 ± 0.03, *p* < 0.01) and lower expression of c-IAP1 (c-IAP1: 0.34 ± 0.06 vs. 0.85 ± 0.10, *p* < 0.01) than mice in the control group. DAPA reduced the level of cardiac cleaved caspase3 (cleaved caspase3: 0.39 ± 0.17 vs. 0.69 ± 0.18, *p* < 0.01), caspase9 (caspase9: 0.36 ± 0.06 vs. 0.62 ± 0.10, *p* < 0.01), and Cyt-C (Cyt-C: 0.45 ± 0.07 vs. 0.81 ± 0.06, *p* < 0.01) while increasing the expression of c-IAP1 (c-IAP1: 0.61 ± 0.13 vs. 0.34 ± 0.06, *p* < 0.01) expression compared with their expression in the METH group (Figures 5E–I). The expression of caspase9 and Cyt-C in the DAPA group increased, while the expression of c-IAP1 in the DAPA group decreased compared with their expression in the control group (all *p* < 0.01). Immunocytochemistry analysis of the expression of cleaved caspase3 and GRP 78 showed that mice in the METH group had significantly higher expression of cleaved caspase3 and lower expression of GRP78 than mice in the control group. DAPA reduced cardiac cleaved caspase3 expression but increased GRP78 expression compared with their expression in the METH group (Figures 5A–D). Therefore, our results revealed that DAPA reduces cardiomyocyte apoptosis through the Cyt-C/caspase3 pathway.

## Discussion

This study explored the preventive effect of DAPA on METH-induced cardiomyopathy in a mouse model. The major findings were as follows: 1) 14-week METH exposure led to impaired cardiac function and mitochondrial injury in a mouse model; 2) DAPA treatment reduced the METH-induced decline in cardiac function and mitochondrial impairment; 3) the protective effects of DAPA could be attributed to the inhibition of cardiac oxidative stress and decreased cardiomyocyte apoptosis through the Cyt-C/caspase3 mitochondrial pathway.

METH exhibits various adverse effects on the cardiovascular system, leading to cardiomyopathy and reduced heart function (Yeo et al., 2007; Sadeghi et al., 2012). In an autopsy report of METH-related deaths attributed solely to METH toxicity, 37.1% cases showed cardiomegaly (Darke et al., 2018). METH-associated cardiomyopathy is characterized by severe heart failure and altered cardiac function. In our study, a 14-week chronic METH exposure mouse model was established, and it demonstrated that long-term METH exposure impaired cardiac function and induced cardiomyopathy. However, DAPA was effective in restoring LV function when exposed to METH. Mice treated with DAPA showed a significant improvement in LVEF and LVFS and a reduction in LVID. However, ventricular wall thinning was not significantly alleviated. The neurohormones NE and BNP are considered markers of heart failure progression (Neuhold et al., 2010). Our findings indicated that DAPA inhibited METH-induced increase in plasma NE and BNP levels. This result suggests that DAPA therapy could prevent the negative impact of METH on cardiac function and ventricular dilation in mice.

DAPA is a traditional hypoglycemic drug that not only lowers blood glucose levels in diabetes models, but also protects the cardiac tissue in non-diabetes models (Lee et al., 2017). Therefore, the findings of this research could provide insights for future clinical treatments of METH-induced cardiac injury. Our results suggested that DAPA reduces myocardial alterations triggered by METH exposure. However, the key regulator involved in the cardioprotective effect of DAPA needs to be explored.

DAPA reportedly improves cardiac morphology and function in a diabetic cardiomyopathy model and a doxorubicin cardiomyopathy model by enhancing cardiac energy metabolism and decreasing oxidative stress and myocardial cell apoptosis (El-Shafey et al., 2022) (Chang et al., 2022). However, whether DAPA can ameliorate METH-induced oxidative stress has not been studied.

ROS induces cell apoptosis by activating the mitochondrial pathway (Ma et al., 2014). ROS critically contributes to left ventricular dilation and dysfunction induced by METH (Lord et al., 2010). In the present study too, an increase in ROS production in METH-exposed mice was found, which was significantly reduced by DAPA treatment. The expression of MFN2 and FIS1 was evaluated to further assess the function and morphology of mitochondria. MFN2 and FIS1 levels were decreased in the METH group but increased by DAPA treatment. In addition, transmission electron microscopy and JC-1 staining confirmed that DAPA decreased mitochondrial damage caused by METH. These results suggested that DAPA improves mitochondrial function by reducing the level of ROS stimulated by METH.

Apoptosis is a physiological cell death mechanism that can lead to many cardiac disorders such as heart failure when induced by a pathological signal (Narula et al., 2006). METH induces Bcl-2/Bax-mediated apoptosis in endothelial cells (Cai et al., 2016). DAPA has been demonstrated to protect against apoptosis in ischemia-reperfusion models by inhibiting caspase3 activation (Tanajak et al., 2018). It also downregulates key molecules in the apoptosis pathway in a diabetic cardiomyopathy model (El-Shafey et al., 2022). However, the effect of DAPA on METH-induced cardiomyocyte apoptosis has not been revealed. In our study, TUNEL staining of the myocardium showed that the percentage of cell apoptosis in the METH group was significantly higher than that in the control group, which was reduced by DAPA treatment. The mitochondrial apoptosis pathway is an intrinsic pathway mediated by Cyt-C, leading to apoptosome formation. The apoptosome then activates caspase9 and caspase3 to initiate an apoptotic protease cascade (Xiong et al., 2014; Zhou et al., 2015). The inhibitor of apoptosis protein family of molecules inhibits apoptosis by suppressing caspase activity (Kulathila et al., 2009; Burke et al., 2010). In our study, DAPA reduced the METH-induced increase in levels of Bax, cleaved caspase3, caspase9, and Cyt-C, while reducing the METH-induced decrease in levels of Bcl-2 and cIAP1. GRP78 is an endoplasmic reticulum stress-related protein that protects against apoptosis (Rao et al., 2002). In our study, DAPA reduced the METH-induced increase in GRP78 expression. These results suggested that DAPA reduces apoptosis in METH-exposed mice through the mitochondrial and endoplasmic reticulum stress pathway.

## Study Limitations

There were several limitations in the present study. First, the underlying molecular mechanisms of DAPA and METH still need to be verified *in vitro*. Second, our results showed the preventive effect of DAPA for METH-induced cardiomyopathy, but we did not investigate the effects of DAPA on the animal model after METH-induced cardiomyopathy. Third, we mainly explored the protective effect of DAPA on alleviating oxidative stress and apoptosis but did not explore the mechanism of myocardial fibrosis. Finally, this study provides a preliminary confirmation of the therapeutic effect of DAPA on the cardiac function decline caused by METH through comparisons between the METH group and the METH + DAPA group but does not further explore the pharmacological effect of DAPA by setting the DAPA group alone.

## Conclusion

The present study demonstrated that DAPA inhibits oxidative stress, mitochondrial damage, and cardiomyocyte apoptosis and improves cardiac function after METH exposure. Thus, DAPA might be a promising candidate for treating METH-related cardiomyopathy.

## Data Availability

The original contributions presented in the study are included in the article/Supplementary Material, further inquiries can be directed to the corresponding author.

## Abbreviations

METH, methamphetamine; DAPA, dapagliflozin; LVEF, left ventricular ejection fraction; LVFS, left ventricular fraction shortening; LVIDd, left ventricular internal diameter at end-diastole; LVIDs, left ventricular internal diameter at end-systolic; LVPWd, left ventricular posterior wall thickness at end diastole; LVPWs, left ventricular posterior wall thickness at end systole; NE, noradrenaline; BNP, B-type natriuretic peptide; ROS, reactive oxygen species; MFN2, itofusin2; FIS1, ission1; TUNEL, terminal deoxynucleotidyl transferase dUTP nick-end labeling; Bax, Bcl-associated X; Bcl-2, B-cell lymphoma-2; Cyt-C, cytochrome C; cIAP1, inhibitor of apoptosis 1.
