# Supplementary figures and images for: Dapagliflozin Protects Methamphetamine-Induced Cardiomyopathy by Alleviating Mitochondrial Damage and Reducing Cardiac Function Decline in a Mouse Model
Source: Front Pharmacol. 2022 Jul 7;13:925276. doi: 10.3389/fphar.2022.925276 (PMC9301370; doi:10.3389/fphar.2022.925276)

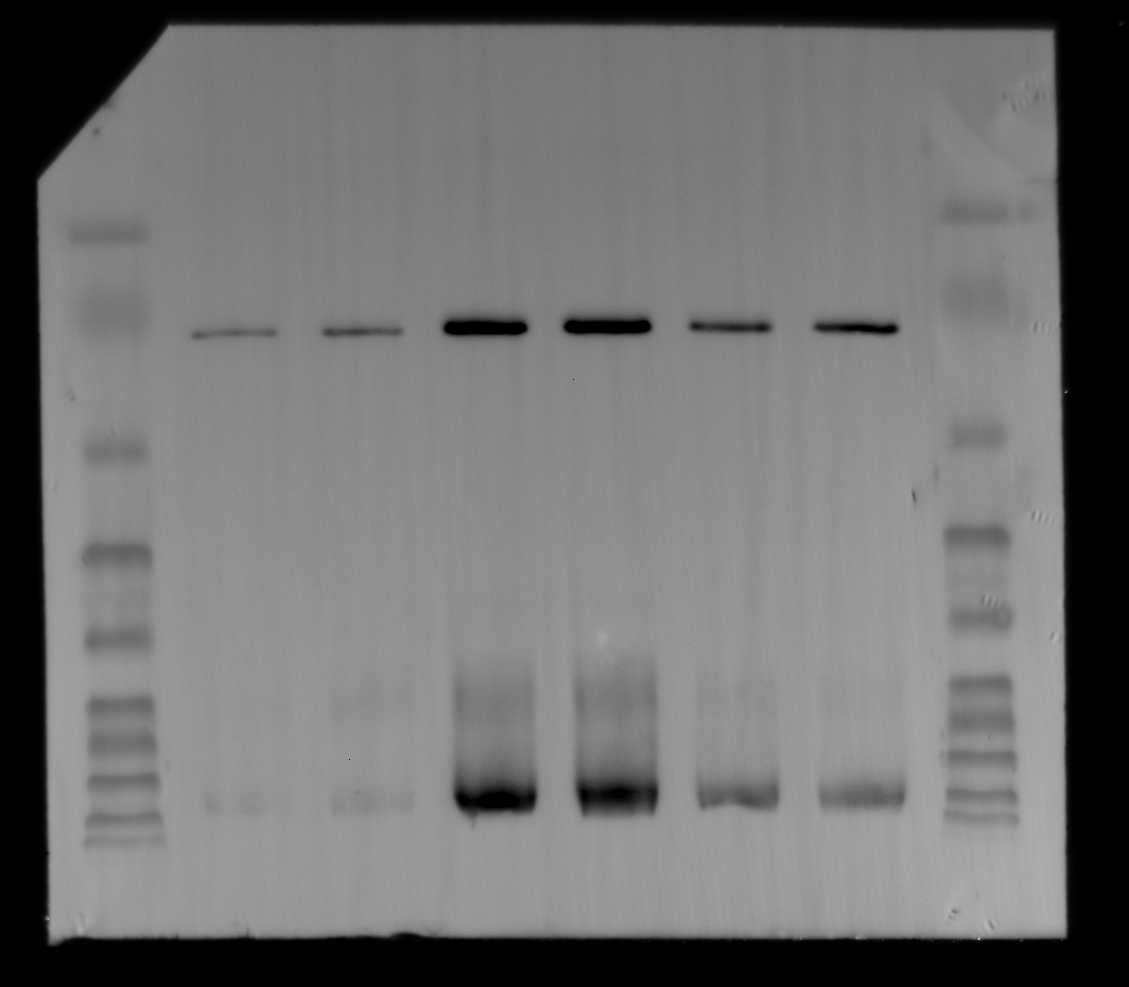

Supplement: Supplementary file 1 [file DataSheet1.ZIP › raw western blot/BAX .tif]

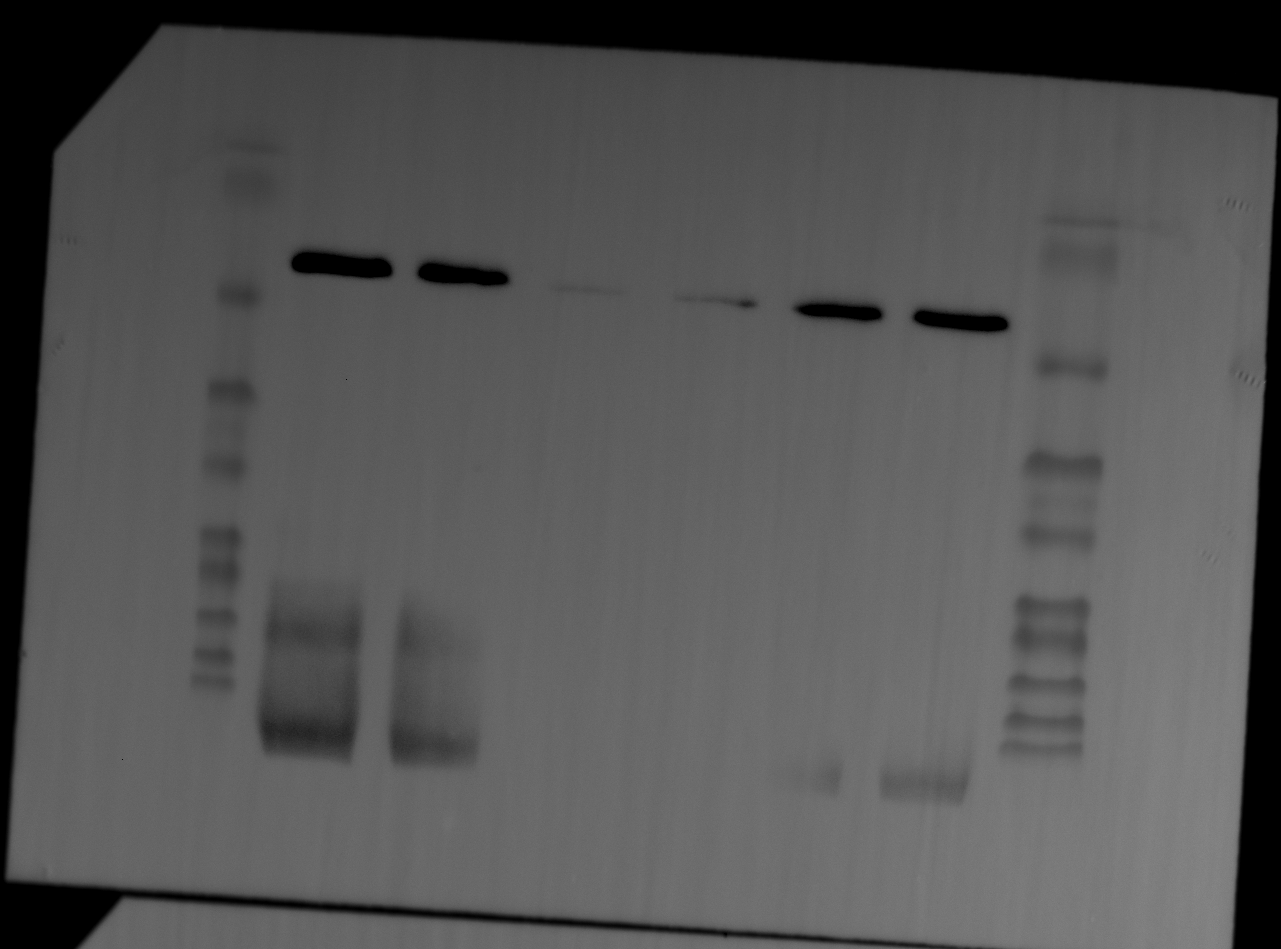

Supplement: Supplementary file 1 [file DataSheet1.ZIP › raw western blot/Bcl-2 .tif]

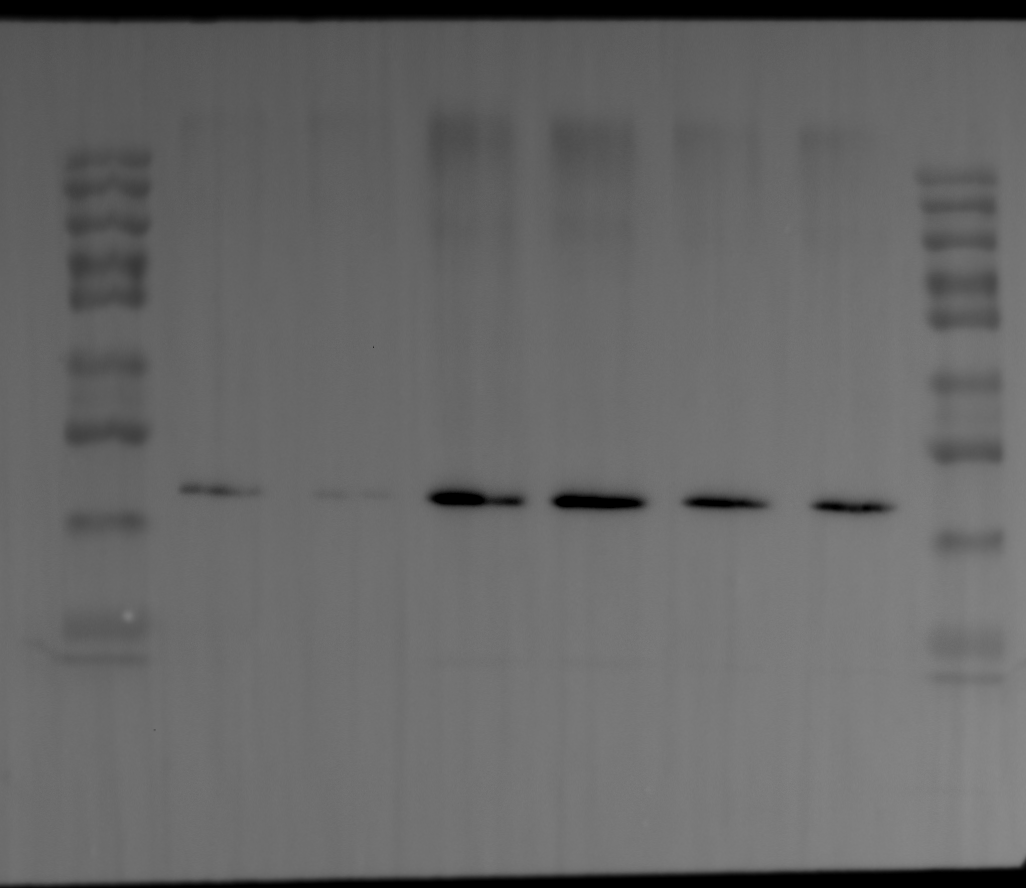

Supplement: Supplementary file 1 [file DataSheet1.ZIP › raw western blot/Caspase9 .tif]

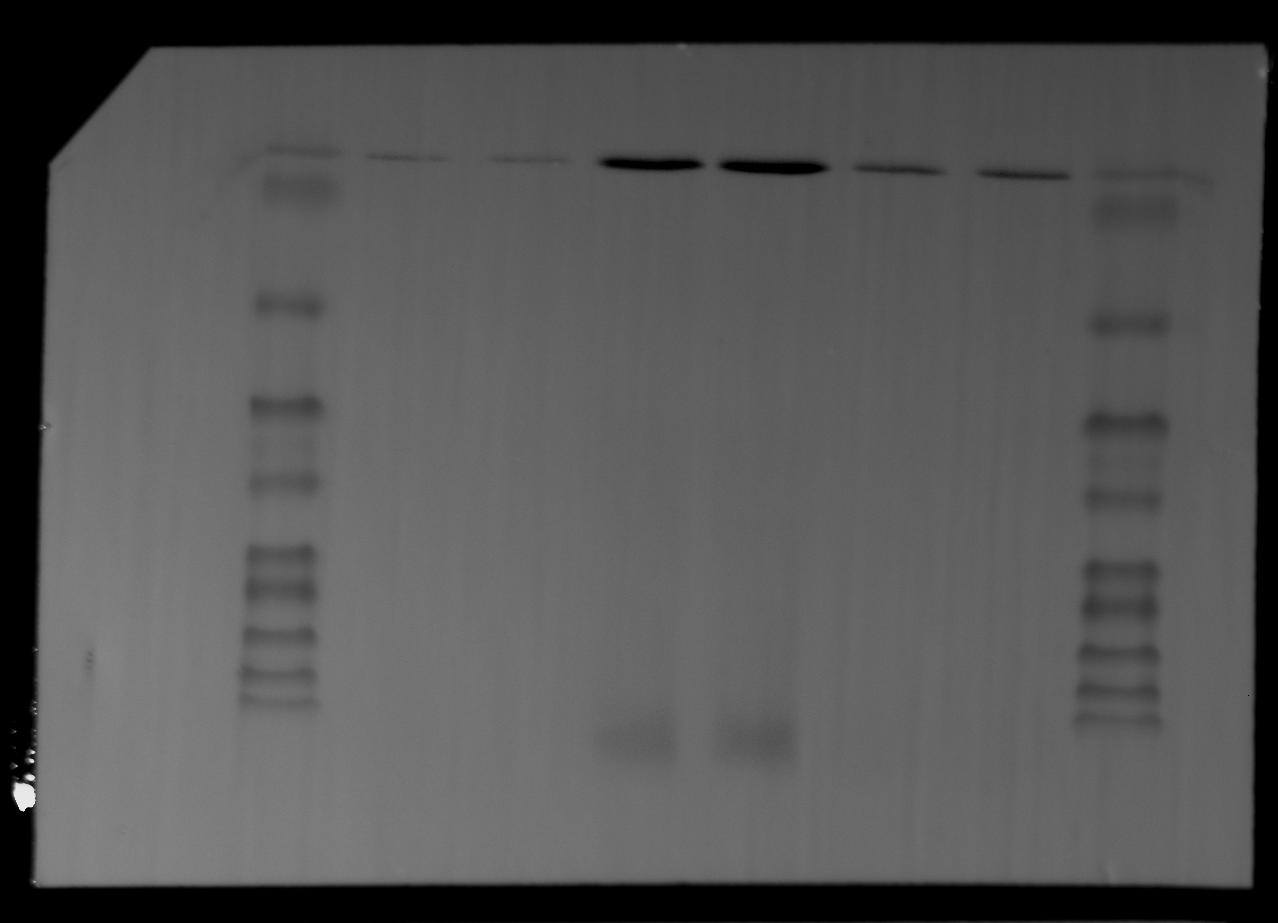

Supplement: Supplementary file 1 [file DataSheet1.ZIP › raw western blot/c-Caspase3 .tif]

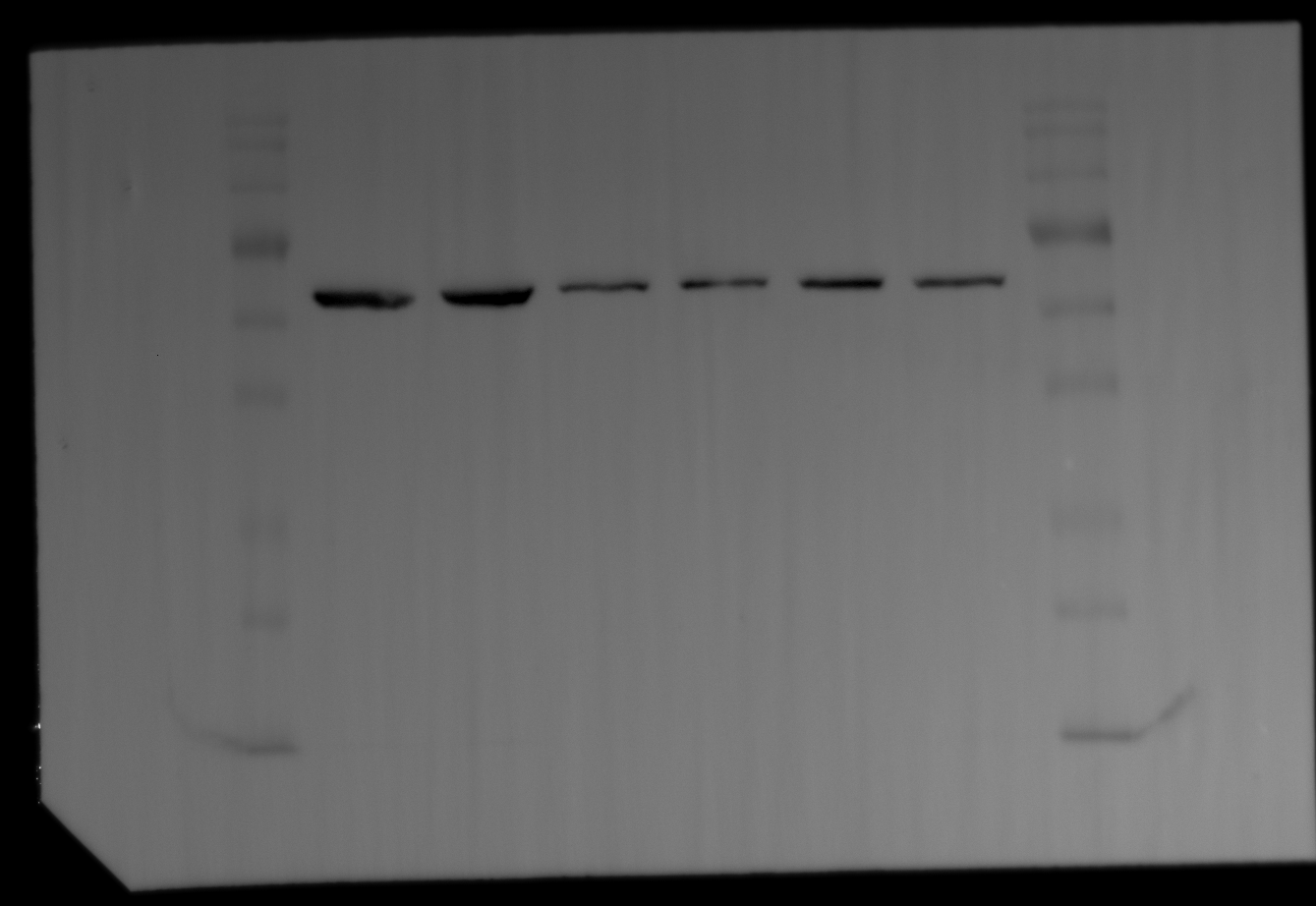

Supplement: Supplementary file 1 [file DataSheet1.ZIP › raw western blot/c-IAP1 .tif]

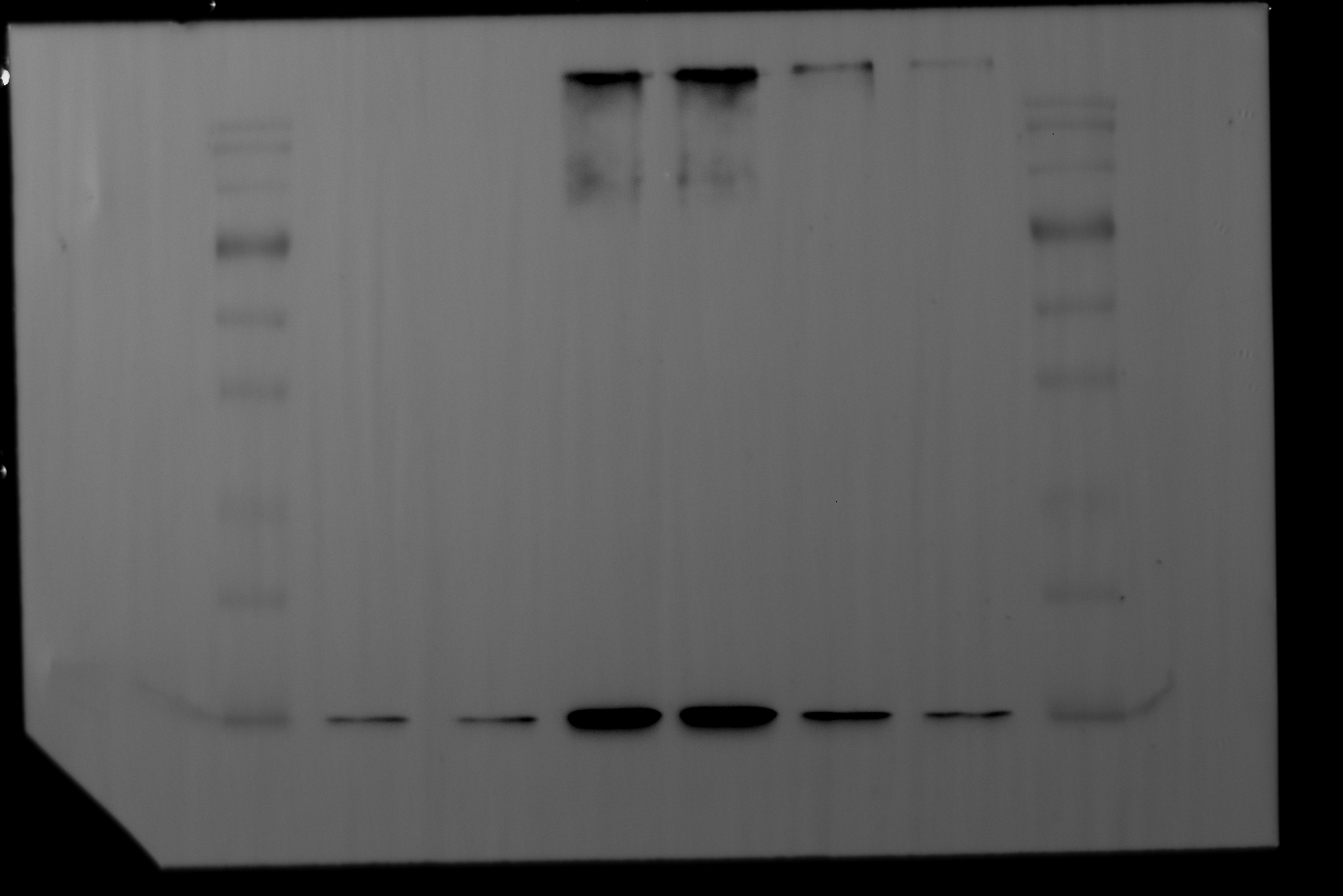

Supplement: Supplementary file 1 [file DataSheet1.ZIP › raw western blot/cyt-c .tif]

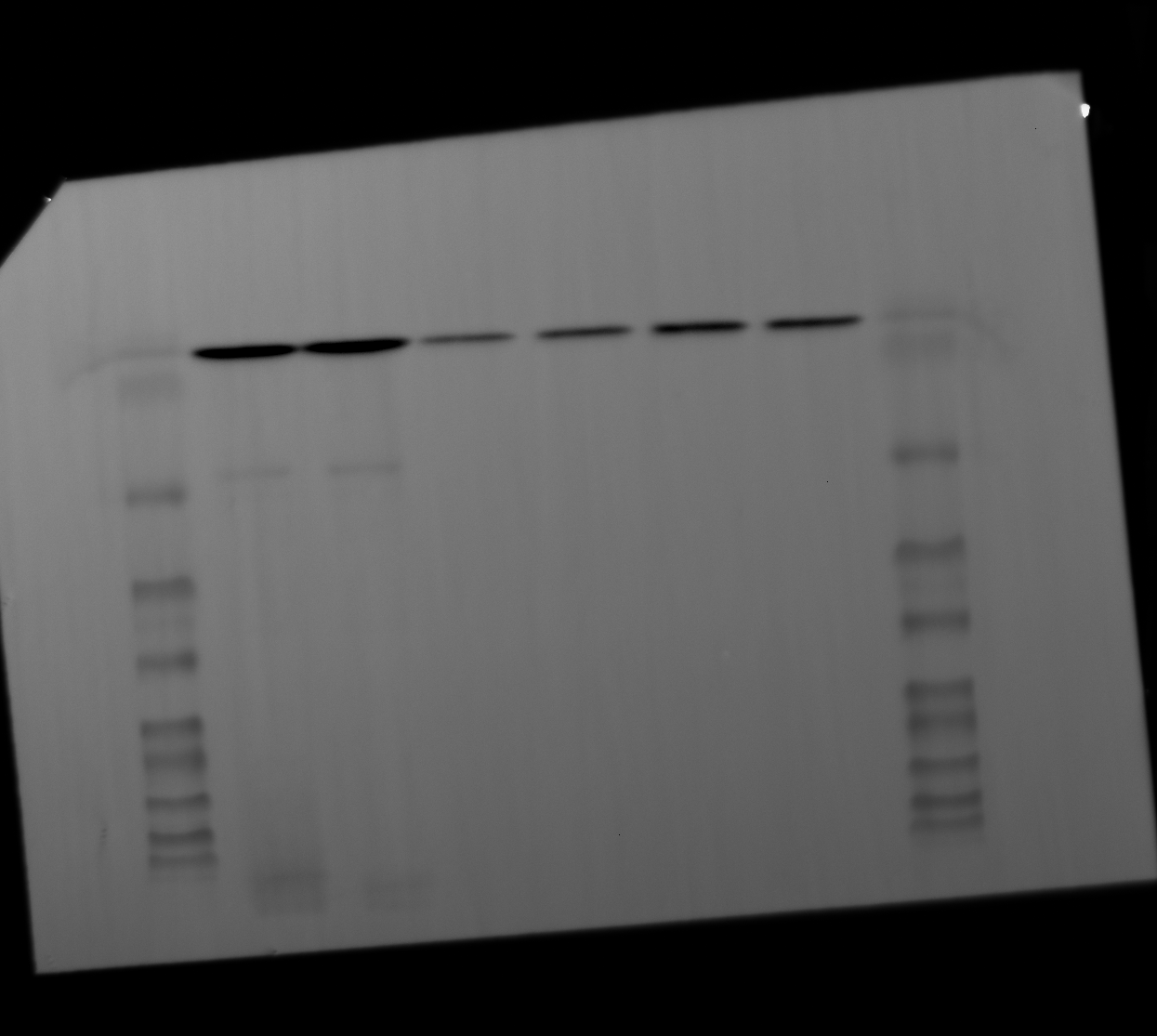

Supplement: Supplementary file 1 [file DataSheet1.ZIP › raw western blot/FIS1 .tif]

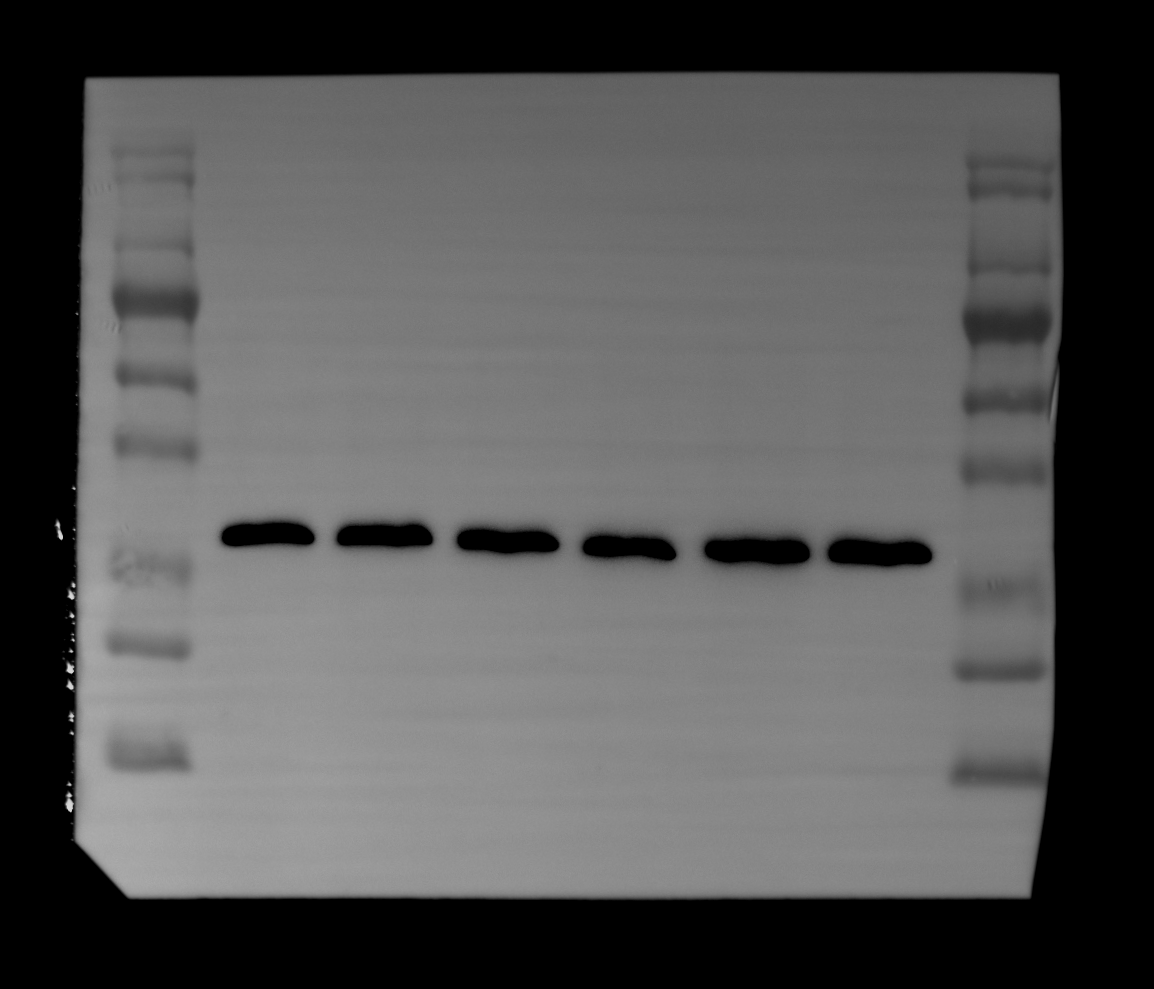

Supplement: Supplementary file 1 [file DataSheet1.ZIP › raw western blot/GAPDH 2.tif]

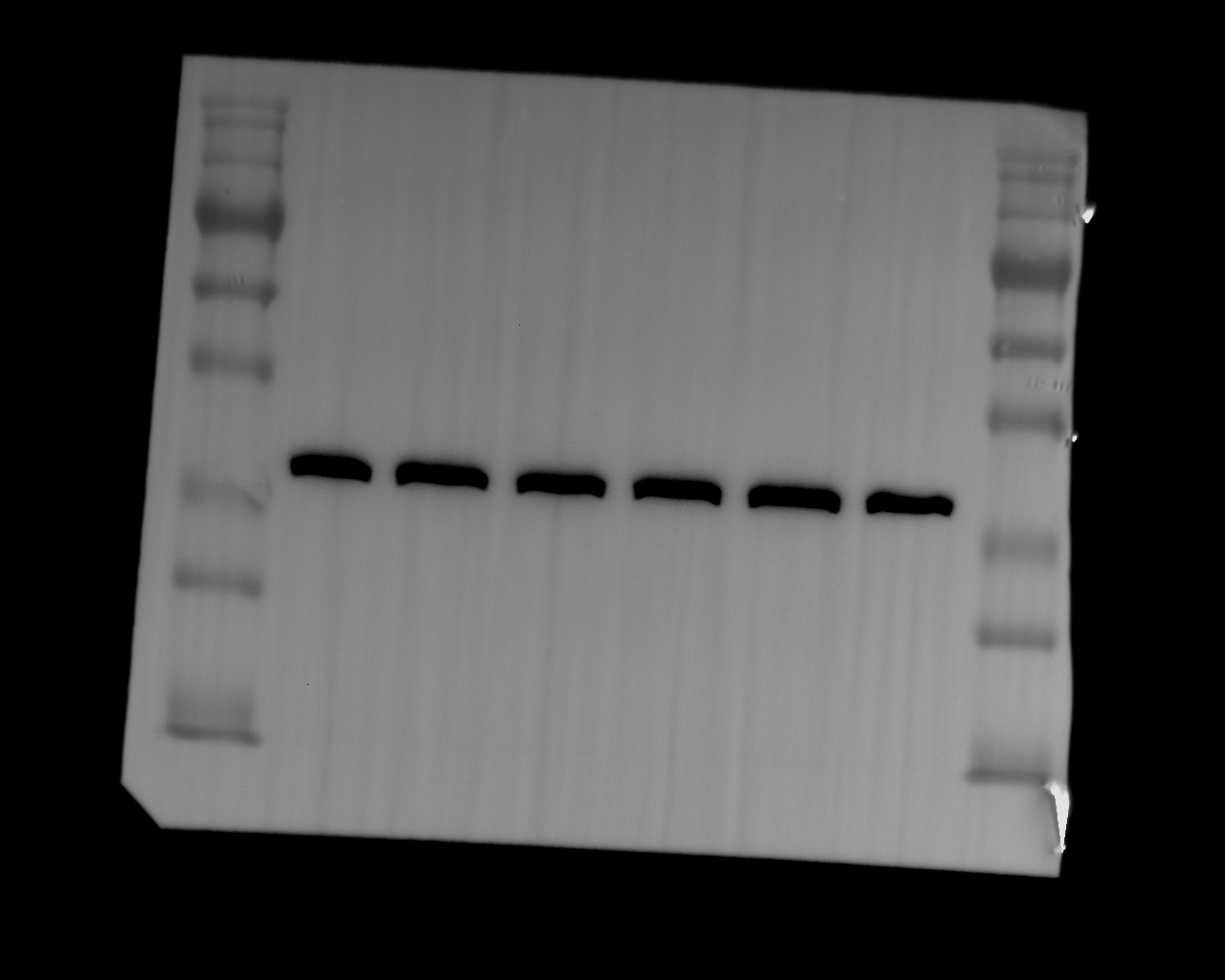

Supplement: Supplementary file 1 [file DataSheet1.ZIP › raw western blot/GAPDH 3.tif]

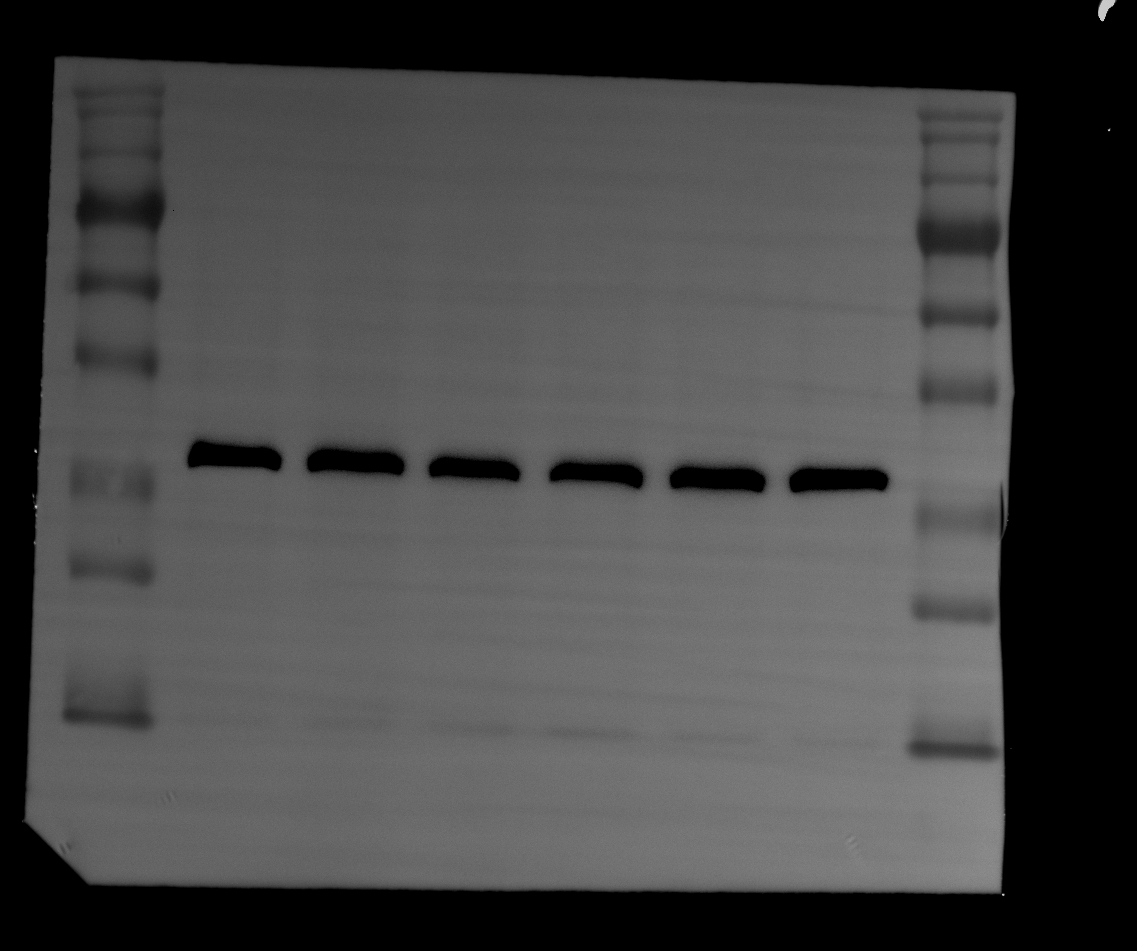

Supplement: Supplementary file 1 [file DataSheet1.ZIP › raw western blot/GAPDH 4.tif]

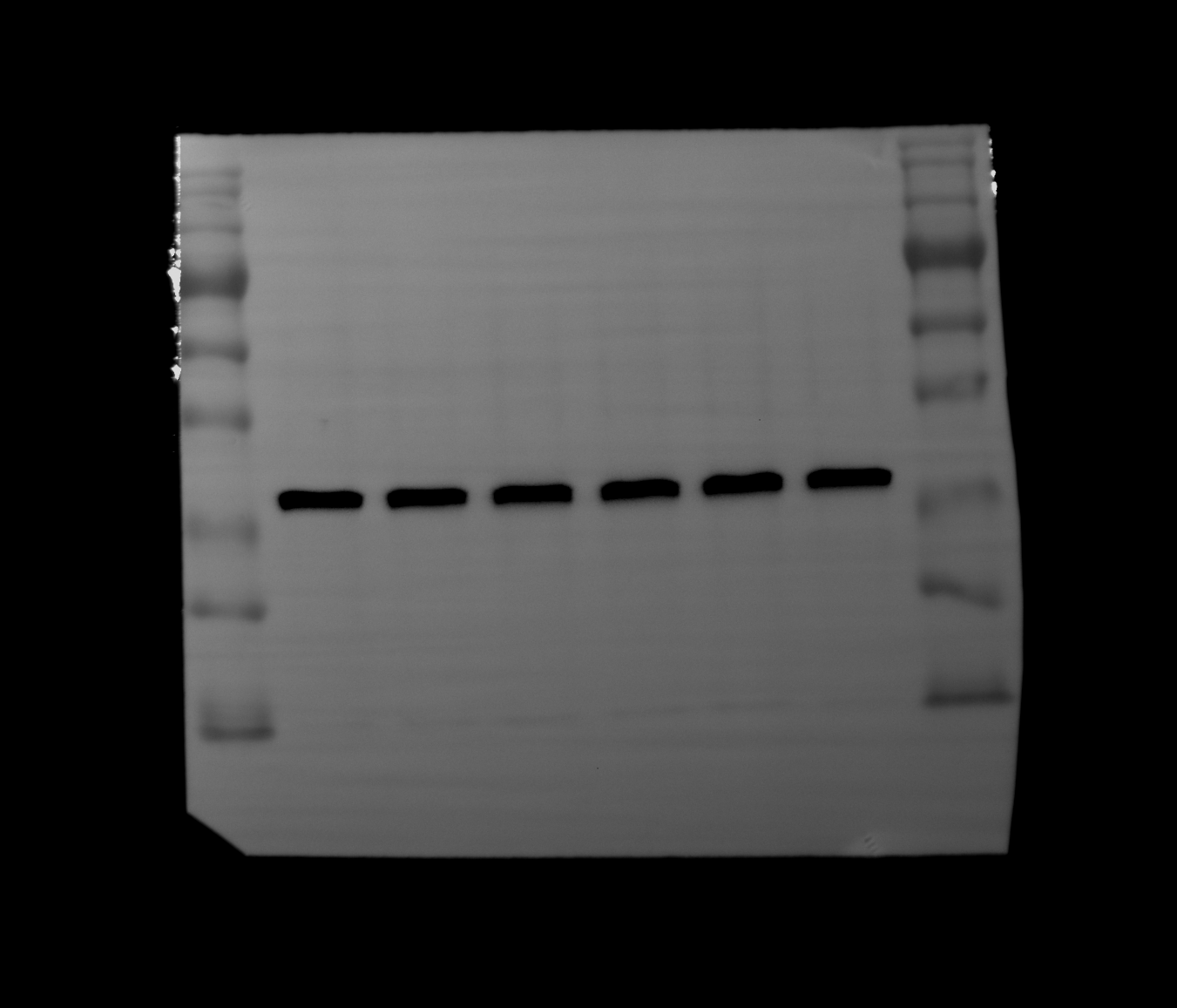

Supplement: Supplementary file 1 [file DataSheet1.ZIP › raw western blot/GAPDH 5.tif]

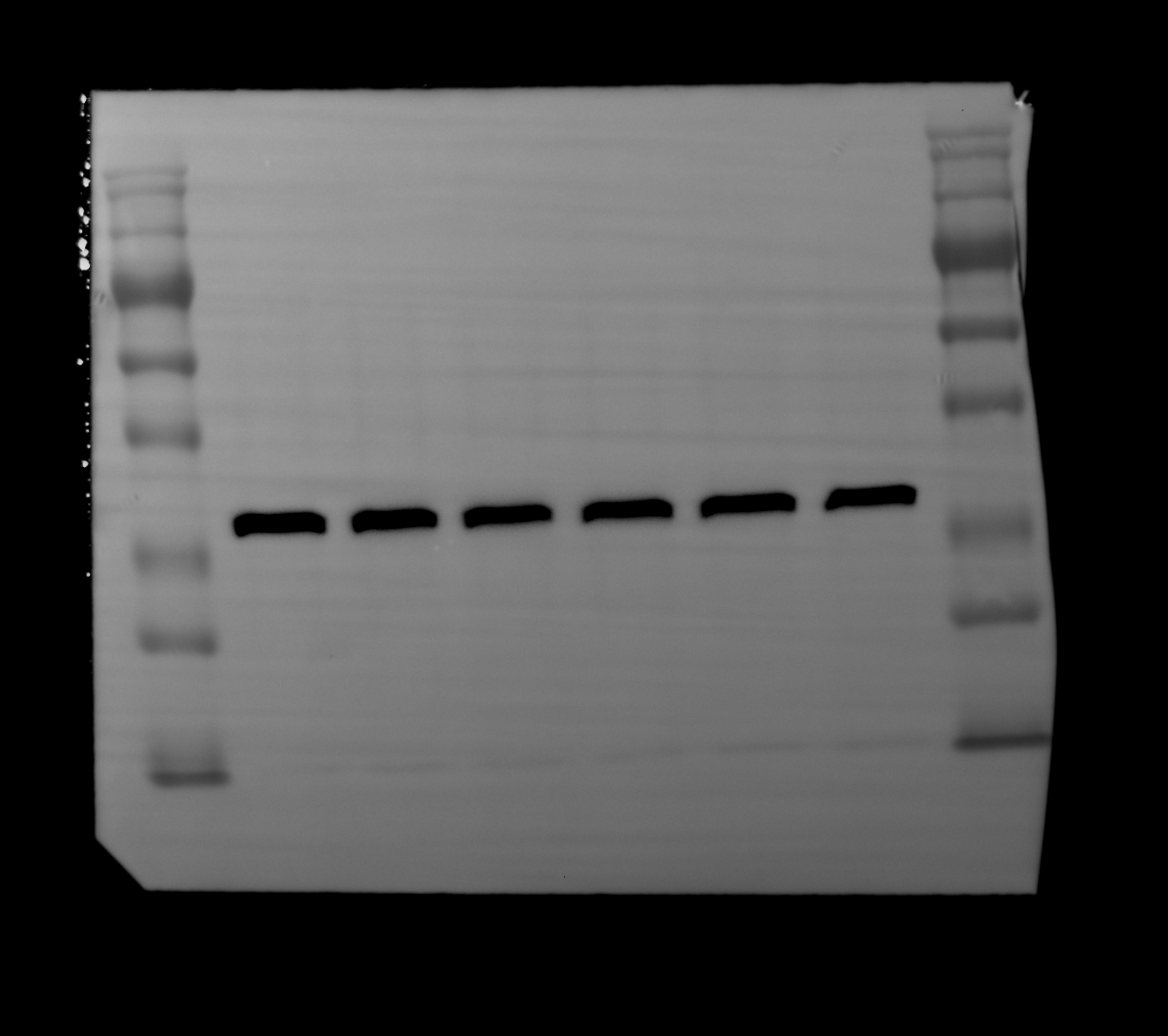

Supplement: Supplementary file 1 [file DataSheet1.ZIP › raw western blot/GAPDH 1.tif]

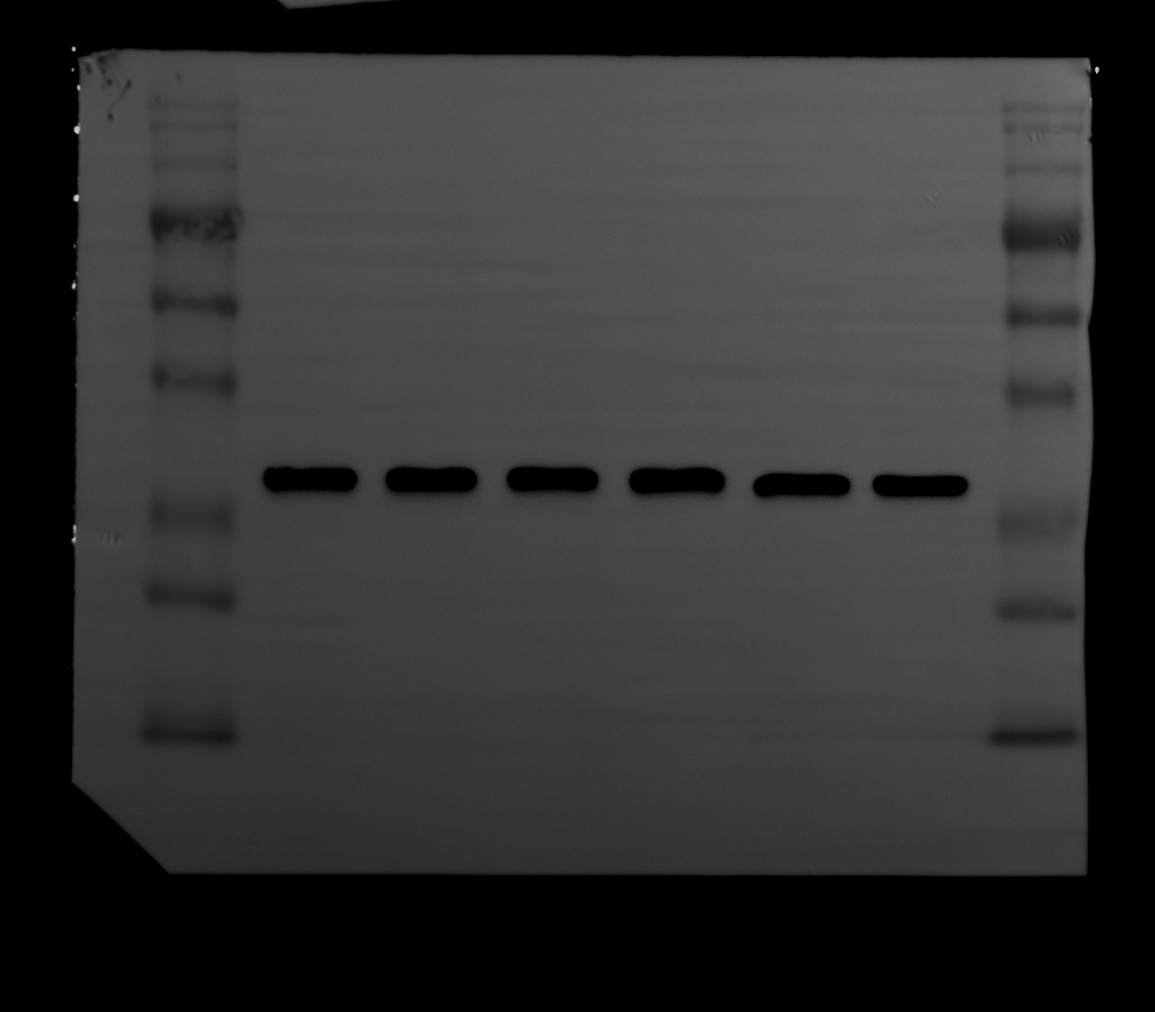

Supplement: Supplementary file 1 [file DataSheet1.ZIP › raw western blot/GAPDH 6.tif]

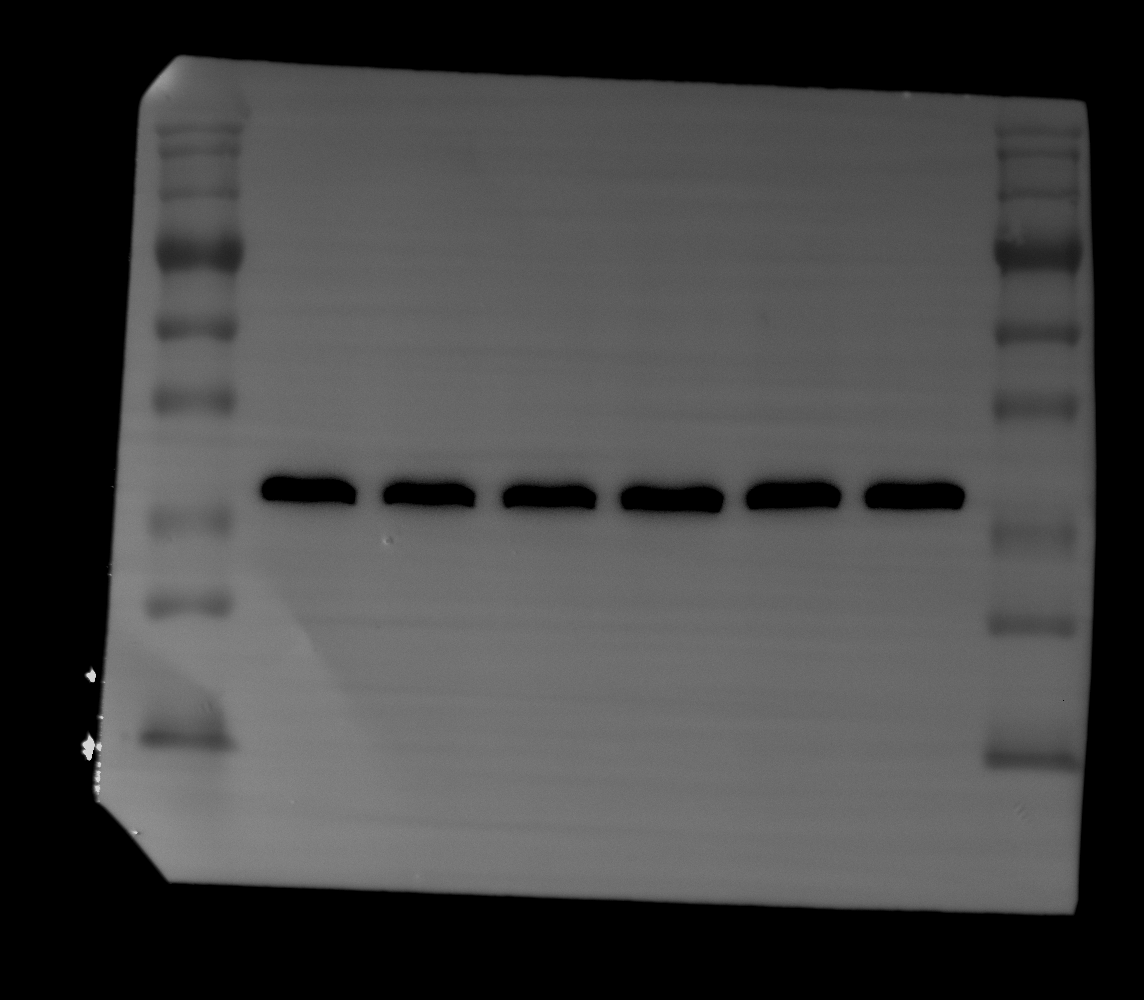

Supplement: Supplementary file 1 [file DataSheet1.ZIP › raw western blot/GAPDH 7.tif]

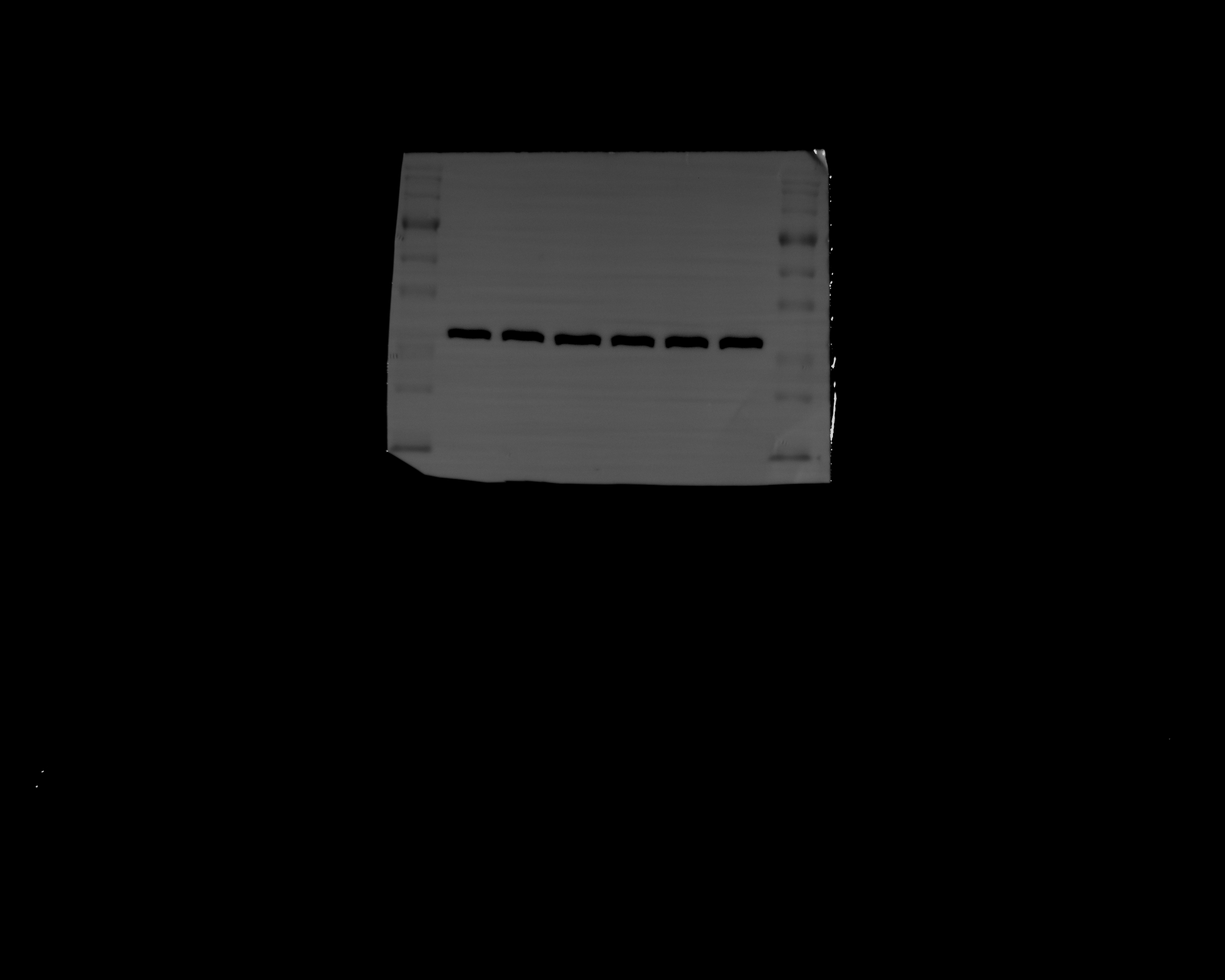

Supplement: Supplementary file 1 [file DataSheet1.ZIP › raw western blot/GAPDH 8.tif]

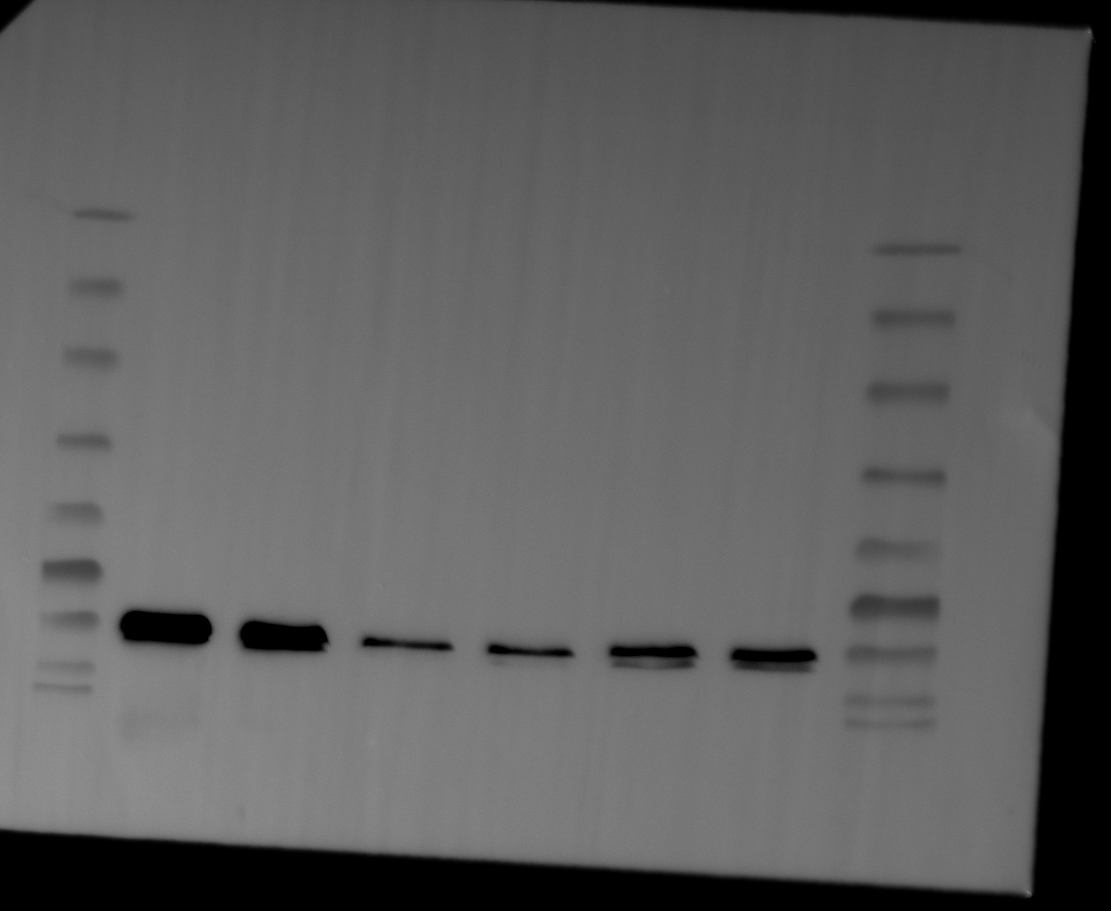

Supplement: Supplementary file 1 [file DataSheet1.ZIP › raw western blot/MFN2 .tif]
